# From Shallow to Full Wrapping: Geometry and Deformability Dictate Lipid Vesicle Internalization

**DOI:** 10.1021/acs.nanolett.5c04322

**Published:** 2025-11-05

**Authors:** Stijn van der Ham, Alexander Brown, Halim Kusumaatmaja, Hanumantha Rao Vutukuri

**Affiliations:** † Active Soft Matter and Bio-inspired Materials Lab, Faculty of Science and Technology, MESA+ Institute, 3230 University of Twente, 7500 AE Enschede, The Netherlands; ‡ Department of Physics, 3057 Durham University, Durham DH1 3LE, U.K.; § Institute for Multiscale Thermofluids, School of Engineering, The University of Edinburgh, Edinburgh EH9 3FB, U.K.

**Keywords:** Membrane wrapping, Giant unilamellar vesicles, Endocytosis, Drug delivery

## Abstract

The deformability and adhesion of vesicles critically influence their engulfment by lipid membranes, a process that is central to endocytosis, viral entry, drug delivery, and intercellular transport. We developed a versatile experimental system of giant unilamellar vesicles (GUVs) that interact via depletion-induced adhesion. Combining experiments with continuum simulations, we construct a state diagram identifying conditions for the endo- and exocytic engulfment of small GUVs by larger ones. Leveraging full 3D confocal reconstructions of vesicle curvature, we experimentally quantify the bendocapillary length, a scale governing the competition between membrane bending and adhesion. When the vesicle size exceeds this length, wrapping is governed by geometry. In contrast, near this scale, deformability controls transitions between shallow, deep, and fully wrapped states, suppressing full engulfment. Finally, we demonstrate on-demand, light-induced switching between wrapping states using photoresponsive lipids. These results establish a mechanical criterion for vesicle engulfment and provide a tunable platform for studying soft cargo uptake.

## Graphical abstract

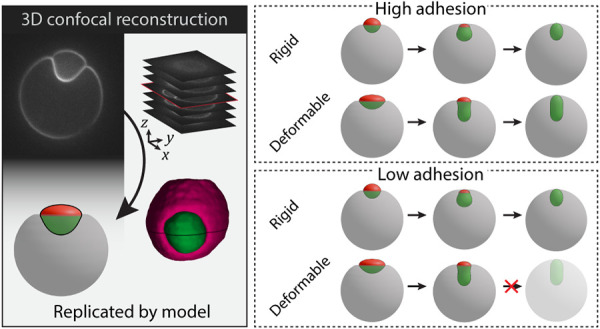

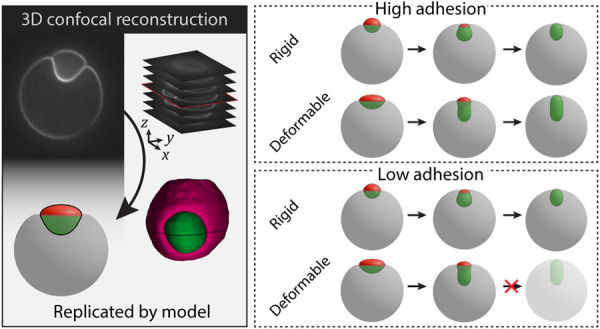

Vesicle engulfment by lipid membranes is a fundamental mechanism by which cells internalize material, enabling cargo exchange across cellular membranes and playing essential roles in intercellular communication, transport, and immune responses.^1^ In biological systems, extracellular vesicles (EVs) transport molecular cargo between cells, while viruses exploit similar mechanisms to gain entry into host cells.^1–4^ In therapeutic contexts, engineered liposomes mimic these mechanisms for targeted drug delivery.^5–7^ While some vesicles cross membranes via direct fusion, many follow wrapping-based pathways such as endocytosis or phagocytosis.^2,8,9^ Understanding the physical principles underlying these processes is essential for interpreting cellular behavior and developing advanced vesicle-based technologies.

Most studies to date have focused on the engulfment of rigid particles,^10–17^ where membrane deformation occurs around a fixed particle shape. In contrast, vesicles are deformable, allowing for mutual reshaping during engulfment. Theoretical studies on the engulfment of soft objects by membranes predict that deformability can stabilize partially wrapped states,^18^ inhibit full engulfment,^19^ and drive mutual shape remodelling.^20^ These objects can be distinguished by their deformation modes. For instance, in condensate droplets, soft particles, and lipid vesicles, these modes are respectively determined by droplet surface tension, particle elasticity, and vesicle bending deformation.^21–24^ In parallel, experimental studies on nanoparticles demonstrated that particle elasticity affects cellular uptake and circulation time.^25,26^ Vesicle–vesicle adhesion studies have further shown that contact morphologies between similarly sized vesicles result from a balance between bending elasticity, membrane tension, and adhesion strength.^27–31^ However, a systematic experimental investigation into the role of deformability in lipid vesicle engulfment remains elusive.

To address this gap, we develop a model system using giant unilamellar vesicles (GUVs),^32^ in which small vesicles interact with larger ones via depletion-driven adhesion. We combine experiments and theory to examine how adhesion and membrane bending govern engulfment and characterize this using the bendocapillary length, a characteristic scale at which bending and adhesion energies balance. When the small vesicle is much larger than this length, wrapping is primarily governed by the geometry. In contrast, when the vesicle size is comparable to this scale, the deformability strongly influences transitions between partially and fully wrapped states.

## Experimental Realization of Vesicle–Vesicle Engulfment

To experimentally investigate vesicle engulfment, we use a biomimetic system where large GUVs engulf smaller ones, mimicking cellular vesicle uptake (Movie S1). GUVs of varying sizes were prepared using the droplet transfer method^35^ see (Supporting Information Section 1). Adhesion between vesicles was induced by suspending them in a solution containing polyacrylamide (0.25 wt %), a nonadsorbing polymer that gives rise to depletion-driven adhesion (see Supporting Information Section 1). This yields an adhesion energy *E*_ad_ = *wA*_c_ proportional to the contact area *A*_c_, where *w* is the adhesion strength per unit area.^12,16,36,37^

The direction of engulfment can be influenced by adding the polymer to either the outer or inner solution of the GUVs (Figure 1A,B). When the polymer is added to the outer solution, it creates depletion layers outside the GUVs, promoting adhesion to external vesicles and leading to endocytic engulfment (Figure 1C). Conversely, adding polymer to the inner solution generates depletion layers inside the GUVs, which can drive adhesion to internal vesicles and lead to exocytic engulfment (Figure 1D). The direction of engulfment is also reflected in the fluorescence signal of the GUV membranes (Figure 1C,D). Membrane overlap in the adhered region results in markedly increased fluorescence intensity, appearing as a central peak in the line profile for endocytic engulfment and as an outer peak for exocytic engulfment (Figure 1E,F).

**Figure 1:**
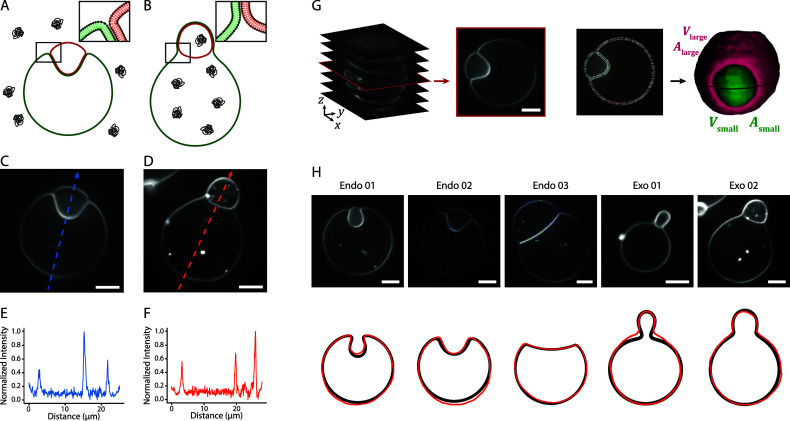
A, B) Schematic representation of endocytic (A) and exocytic (B) engulfment of a small vesicle (red membrane) by a large vesicle (green membrane) where the polymer is added to the outer and inner solution, respectively. C, D) Confocal fluorescence microscopy images of the midplane of partial endocytic (C) and exocytic (D) vesicle engulfment. E, F) Fluorescence intensity profile along the dashed lines from panel C and D. G) 3D reconstruction of vesicles from confocal fluorescence *z*-stacks. Image stacks were segmented using the LimeSeg plugin in FIJI (ImageJ),^33,34^ enabling quantitative extraction of vesicle volume (*V*), surface area (*A*), mean curvature (*M*), and shape. H) The top row shows confocal fluorescence microscopy images of five vesicle pairs that were used for the calibration of *L*_exp_. The bottom row shows the corresponding cross-sectional shape comparison between the experimental (red) and simulated (black) large vesicle morphology for *L*_exp_ = 0.6 μm. Scale bars are 5 μm.

## Modeling the Vesicle–Vesicle Engulfment

To interpret the observed engulfment morphologies, we modeled vesicle–vesicle interactions using energy minimization within a continuum framework (see Supporting Information Section 1), balancing adhesion energy gain and bending energy cost. The model captures the experimental behavior using four dimensionless parameters: the volume ratio of the small and large vesicle, their reduced volumes, and the ratio of the small vesicle size to the bendocapillary length. The volume ratio ϕ is defined as the volume of the small vesicle, *V*_small_, divided by that of the large vesicle, *V*_large_:

1 $$\phi =\frac{V_{small}}{V_{large}}$$

The reduced volume ν_*i*_ (*i* = small, large) quantifies the vesicle deformability and is defined as the ratio between the vesicle’s volume *V*_*i*_ and the volume *V*_*i*,sph_ of a sphere having the same surface area *A*_*i*_:

2 $$\nu_{i}=\frac{V_{i}}{V_{i,sph}}=\frac{3\sqrt{4\pi }V_{i}}{A_{i}^{3/2}}$$

A vesicle with ν = 1 behaves like a rigid spherical particle. As ν decreases, the excess membrane area increases, allowing the vesicle to adopt a broader range of nonspherical shapes, thus increasing its deformability.^38^

Finally, the ratio *R*_small_/*L* characterizes the balance between adhesion and membrane bending forces, where *R*_small_ is given by

3 $$R_{small}={\left(\frac{3}{4\pi }V_{small}\right)}^{1/3}$$

and *L* is defined as

4 $$L=\sqrt{\frac{\kappa }{w}}$$

with κ the bending rigidity of the membrane. For *R*_small_/*L* ≫ 1, adhesion and surface tension forces dominate, while for *R*_small_/*L* ≪ 1, bending rigidity dominates.

We determined ϕ, ν_large_, and ν_small_, by measuring the volume and surface area from 3D confocal microscopy reconstructions using a contour segmentation algorithm (Figure 1G and Supporting Information Section 1). The experimental bendocapillary length was determined by comparing vesicle curvature profiles with model predictions. For five vesicle pairs (Figure 1H), we replicated ϕ, ν_large_, and ν_small_ in simulations, while treating the bendocapillary length *L*_sim_ as a tunable parameter. The *L*_sim_ value for which the simulated vesicle’s curvature best matched the experimental curvature was identified and used to calculate

5 $$L_{exp}=\frac{R_{small,\exp}}{R_{small,sim}}\cdot L_{sim}$$

­(details in Supporting Information Section 1). Averaging across five vesicle pairs yielded *L*_exp_ = 0.6 ± 0.1 μm, which is used throughout our analysis. Cross-section comparisons between experiments and simulations are shown in Figure 1H, demonstrating close agreement.

Although *L*_exp_ should be approximately constant for a given polymer concentration, the vesicle size *R*_small,exp_ varies, causing changes in *R*_small,exp_/*L*_exp_. Therefore, for each vesicle pair, we replicate *R*_small_/*L* in simulations, enabling direct comparison to experiments. This framework allows us to systematically investigate the role of the four dimensionless parameters in vesicle engulfment.

## Endo- and Exocytic Engulfment of Vesicles with Varying Volume Ratio

Combining experiments and continuum simulations, we begin by studying how the direction of engulfment influences vesicle morphology (Figure 2A,B). We explore this behavior by studying vesicle pairs across a range of volume ratios (ϕ = 0.001–0.799), while keeping their reduced volumes approximately constant. Because of experimental variability, we limited our analysis to vesicle pairs with reduced volumes between 0.87 and 0.97 for both large and small vesicles. We employ the same polymer concentration of 0.25 wt % polyacrylamide, which should lead to a constant adhesion strength. However, due to vesicle size variation, the ratio *R*_small_/*L* ranges from 1.8 to 20.

**Figure 2:**
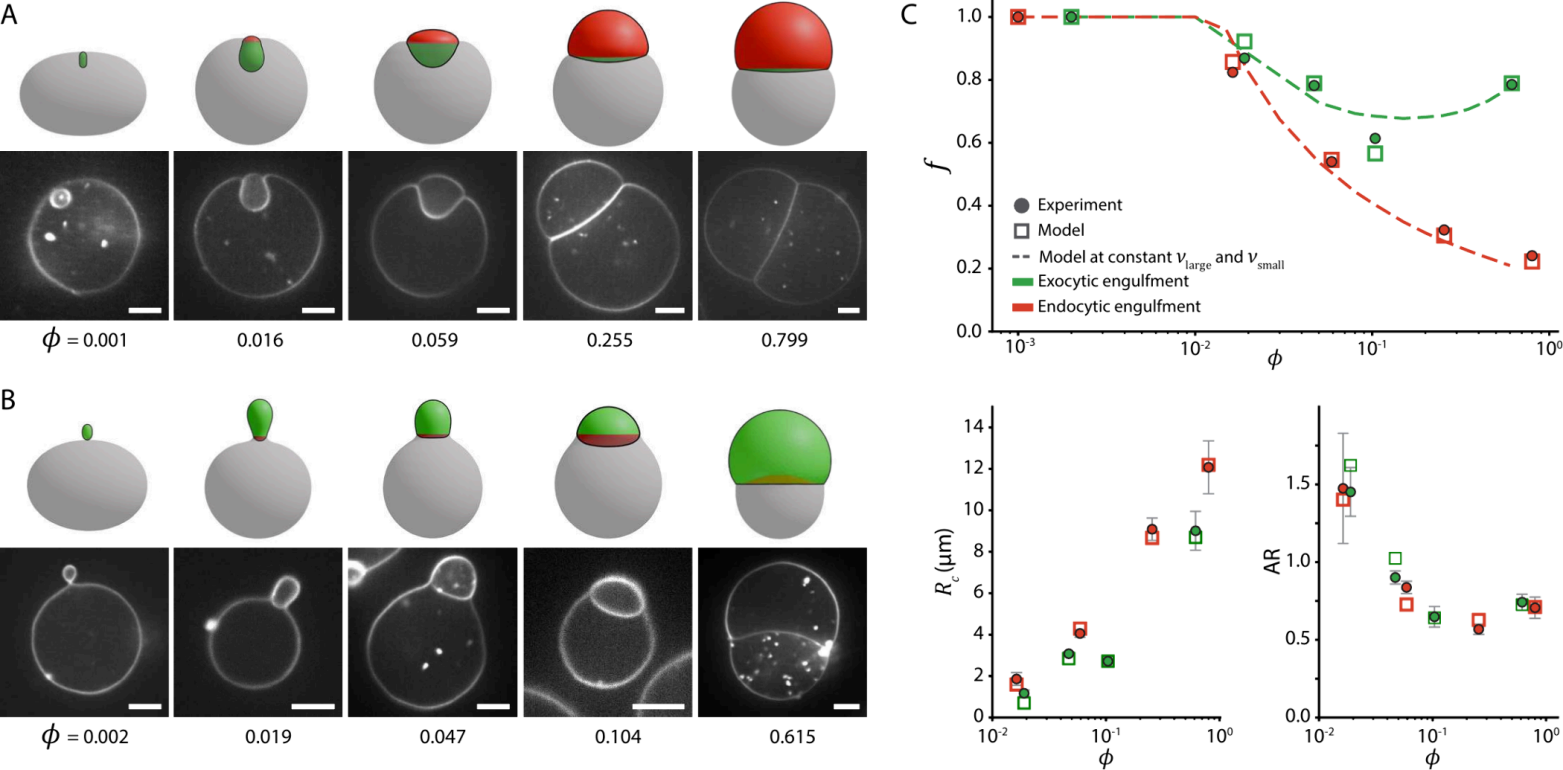
Endo- (A) and exocytic (B) vesicle engulfment as a function of volume ratio ϕ. Confocal fluorescence microscopy images representing equilibrium conformations at various ϕ (indicated below the images). All vesicles had a reduced volume between 0.87 and 0.97 and were recorded at the same adhesion strength (0.25 wt % polyacrylamide). The ratio *R*_small_/*L* ranges from 1.8 to 20. For endocytic engulfment (A), the polymer was present in the outer solution, whereas for exocytic engulfment (B), the polymer was present in the inner solution. The corresponding morphologies, replicated by the model, are shown above the microscopy images. The large vesicle is represented in gray, the small vesicle in red, and the contact area is highlighted in green. Scale bars are 5 μm. C) Quantitative comparison between experiments and simulations for the vesicles shown in panels A (red) and B (green). In all plots, circles denote experimental data and squares denote simulated counterparts. Top panel: wrapping fraction *f* as a function of ϕ. Dashed lines indicate simulated *f*-values for endocytic (ν_large_ = 0.9 and ν_small_ = 0.94) and exocytic (ν_large_ = 0.92 and ν_small_ = 0.92) engulfment, with *R*_small_/*L* = 5 held constant. Bottom panels: Contact-line radius *R*_c_ and small vesicle aspect ratio AR as a function of ϕ for the partially wrapped vesicles. Error bars represent the standard deviation in the experimental data, originating from the variation around the symmetry axis.

During interaction, the membranes’ flexibility drives both vesicles to remodel each other, minimizing the system’s energy. In endocytic engulfment (Figure 2A), the large vesicle forms an invagination at the contact site that deepens with decreasing ϕ. Conversely, in exocytic engulfment (Figure 2B), a protrusion forms around the smaller vesicle. In both cases, the small vesicle undergoes shape changes as it becomes increasingly wrapped, transitioning from an oblate form in the shallow-wrapped state to a prolate geometry in the deep wrapped state. As the wrapping fraction approaches 1, a narrow catenoidal membrane neck forms, creating either an inward-facing bud (endocytic) or an outward-facing bud (exocytic) that fully encapsulates the smaller vesicle.

In the fully wrapped state (leftmost panels of Figure 2A,B), small discrepancies appear between experiments and simulations. Experimentally, large vesicles are slightly flattened, whereas simulations predict a more elongated, prolate shape. This deviation stems from gravity due to a small density mismatch between the inner and outer solutions (osmotically matched sucrose versus glucose), which causes size-dependent flattening of large vesicles.^39^ We further quantified this effect in Supporting Information Section 2.

To further validate our observations, we compared experimental vesicle shapes with model predictions using three independent geometric observables: the wrapping fraction *f* = *A*_c_/*A*_small_, the contact line radius *R*_c_, and the small-vesicle aspect ratio AR (Figure 2C; see Supporting Information Section 3 and Figure S5). The open symbols correspond to simulations matched to the measured dimensionless variables (ϕ, ν_large_, ν_small_, *R*_small_/*L*, where *L*_exp_ = 0.6 μm), while the dashed lines show representative trends obtained for fixed parameter sets. Across both endo- and exocytic regimes, the model and experiments show good quantitative agreement in all three observables, supporting the validity of the mechanical framework.

## Endocytic Vesicle Engulfment and the Role of Vesicle Reduced Volume

While our system enables the study of both endo- and exocytic engulfment (Figure 2), we focus on endocytic uptake to explore how vesicle deformability influences the wrapping morphology. We map vesicle–vesicle shapes as a function of ν_small_ and ν_large_ at an approximately constant volume ratio ϕ (Figure 3A). Here, ν_small_ sets the deformability: even slight deviations from ν_small_ = 1 enable the small vesicle to adapt to the curvature imposed by the large vesicle. In contrast, ν_large_ reflects the excess membrane area available for wrapping and primarily governs the transitions among shallow, deep, and fully wrapped states. Within each regime, ν_small_ shapes the morphology. In the shallow wrapped state, small vesicles with a lower reduced volume appear more oblate, lying flatter against the large vesicle, whereas in the deep wrapped state they transition toward a more prolate form oriented perpendicular to the membrane (Figure 3A). In the fully wrapped state, the small vesicle recovers the morphology of a free vesicle with the same reduced volume but enclosed by the larger vesicle’s membrane. In this regime, minor differences between experiments and simulations arise from size-dependent gravitational flattening (Supporting Information, Section 2).

**Figure 3:**
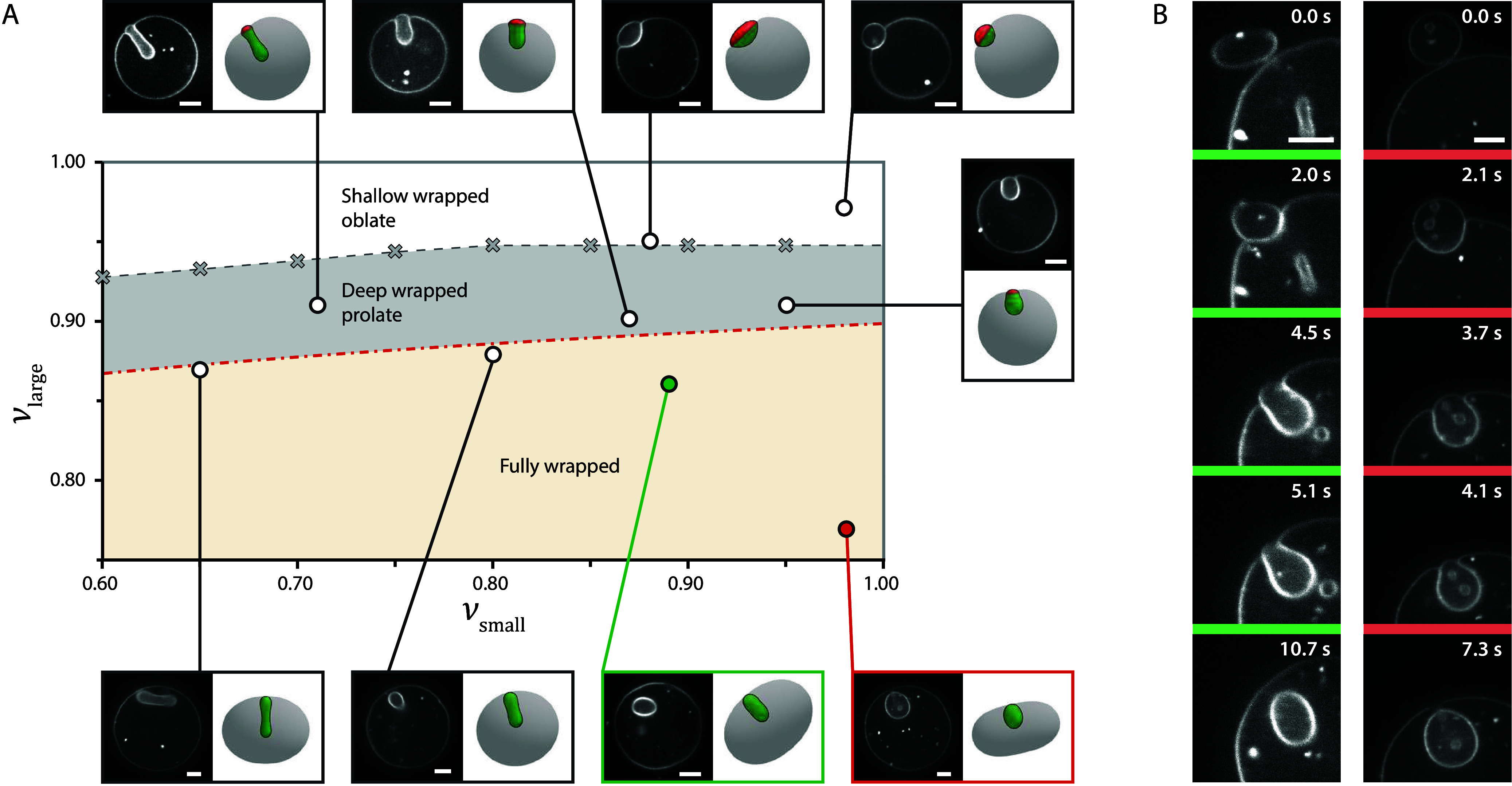
State diagram of endocytic vesicle engulfment as a function of ν_large_ and ν_small_. A) Vesicle-vesicle morphologies as a function of ν_large_ (*y*-axis) and ν_small_ (*x*-axis). Confocal fluorescence microscopy images represent typical equilibrium conformations of vesicle pairs for various reduced volumes. The corresponding morphologies that were replicated by the model are shown alongside the microscopy images. All vesicle pairs had a volume ratio between 0.013 and 0.037 and were recorded at the same adhesion strength (0.25 wt % polyacrylamide). The ratio *R*_small_/*L* ranges from 3 to 7.3. The shaded areas represent shallow wrapped oblate vesicles (white), deep wrapped prolate vesicles (gray), and fully wrapped vesicles (sand). The transition between the shallow and deep wrapped states (gray dashed line) was obtained from simulations (gray crosses). The transition to the fully wrapped state (red dash-dotted line) was calculated with eq 7 using ϕ = 0.016. B) Time series captured with confocal fluorescence microscopy, illustrating the morphology of two vesicles (green: ν_small_ = 0.89 and red: ν_small_ = 0.98 from panel A) as they transition from the free (*f* = 0) to the fully engulfed state (*f* = 1). Scale bars are 5 μm.

The occurence of shallow-wrapped oblates and deep-wrapped prolates reflects the orientational stability predicted for rigid ellipsoids.^13,40^ However, unlike rigid ellipsoids or rods, which reorient during wrapping,^11–13,40^ deformable vesicles can continuously reshape themselves, transitioning from oblate to prolate as the wrapping fraction increases.^19,22^ This dynamic adaptation is further illustrated by the continuous shape adaptations observed during the transition of a small vesicle from the free to fully wrapped state, as shown in Figure 3B and Movies S2 and S3 for two different values of ν_small_, corresponding to the vesicle pairs in Figure 3A marked with green (ν_small_ = 0.89) and red (ν_small_ = 0.98).

## Geometrically Constrained Engulfment

To further characterize the onset of full engulfment, we examine how this transition depends on the vesicle geometry. In the *R*_small_/*L* ≫ 1 regime, the full engulfment transition is determined primarily by the system’s geometry: ϕ, ν_large_, and, to a lesser extent, ν_small_. As the small vesicle becomes wrapped, part of the large vesicle’s excess membrane area is consumed, increasing its sphericity, while the remaining membrane must enclose the combined volume of both vesicles. The effective reduced volume of the large vesicle after wrapping a small vesicle is thus:^17,23^

6 $$\nu_{\gamma }=3\sqrt{4\pi }\frac{V_{large}+V_{small}}{{\left(A_{large}-A_{small}\right)}^{3/2}}$$

which can be rewritten in terms of the dimensionless parameters as (see Supporting Information Section 4):

7 $$\nu_{\gamma }=\frac{1+\phi }{{\left(\nu_{large}^{-2/3}-\phi^{2/3}\nu_{small}^{-2/3}\right)}^{3/2}}$$

The value of ν_γ_ quantifies whether full wrapping is possible: when ν_γ_ ≤ 1, the small vesicle can be fully engulfed, whereas when ν_γ_ > 1, the large vesicle lacks sufficient excess membrane area to fully engulf the small vesicle, resulting in partial wrapping. Interestingly, eq 7 shows that the engulfment transition depends only weakly on ν_small_, since it is scaled by ϕ^2/3^, which is small in our study (ϕ < 0.1). This weak dependence highlights that in this regime the small vesicle deformability plays a limited role.

We constructed a state diagram mapping the wrapping outcome as a function of ϕ and ν_large_ (Figure 4A), combining experiments, simulations, and the theoretical transition from eq 7. The narrow shaded region representing ν_small_ = 0.7 to 1 confirms the weak influence of the small vesicle deformability. Experimental data spanning *R*_small_/*L* = 1.8 to 11 align well with the predicted transition, indicating that even modest increases beyond *R*_small_/*L* = 1 are sufficient to reach the geometry-dominated regime. Simulations at *R*_small_/*L* = 10 closely match the analytical prediction, supporting this conclusion.

**Figure 4:**
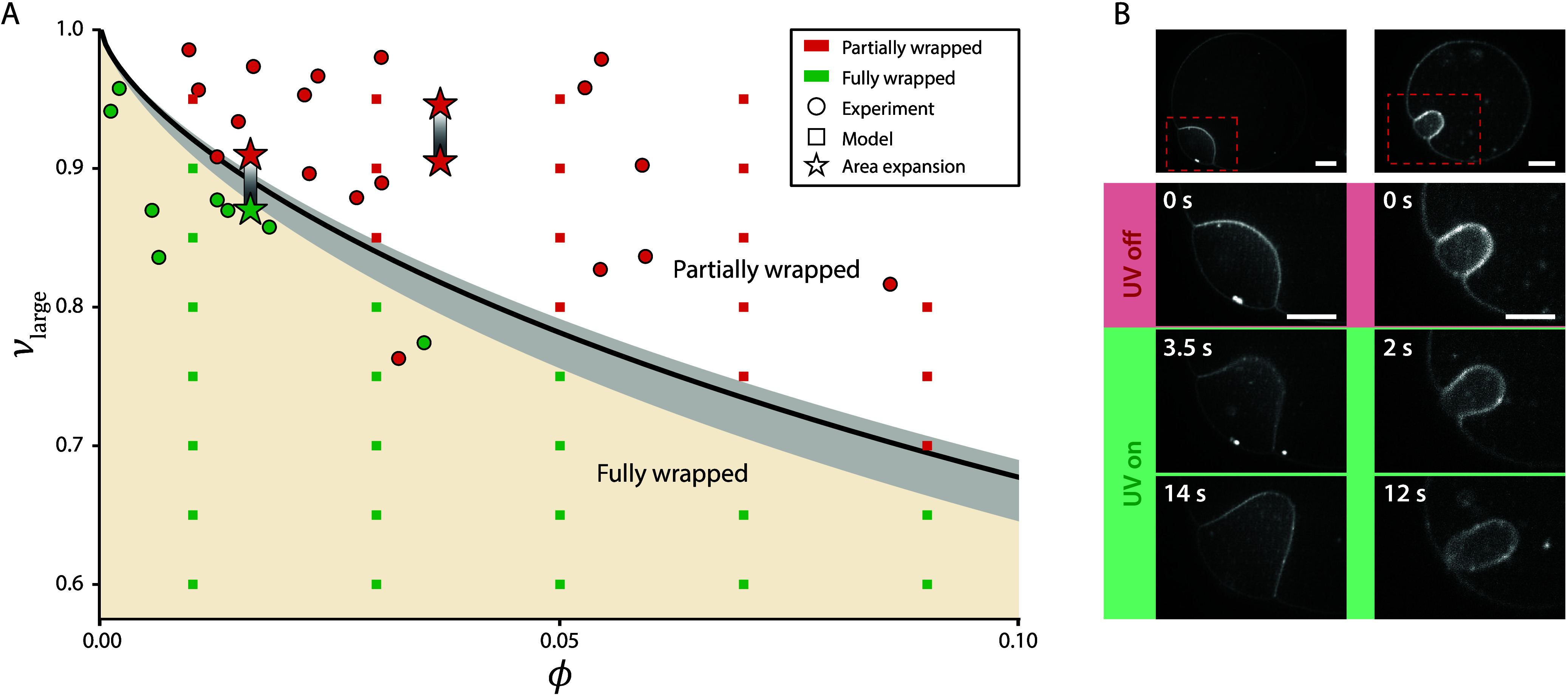
A) State diagram of the partially and fully wrapped state as a function of ν_large_ and ϕ. The black line gives the theoretical transition between the partially (red) and fully wrapped (green) state based on eq 7. The gray shaded area indicates the influence of ν_small_ on this transition. Based on the available experimental data, we show a lower and an upper bound of ν_small_ = 0.7 and 1.0, respectively, and the black line representing ν_small_ = 0.9. Experimental and simulated data points are plotted as circles and squares, respectively. For the simulated data, *R*_small_/*L* = 10 and ν_small_ = 0.9, while for the experimental data *R*_small_/*L* ranges from 1.8 to 11 and ν_small_ ranges from 0.65 to 0.99. Area expansion estimates corresponding to panel B are given by pairs of stars, where the top star corresponds to the vesicle pair before UV illumination, and the bottom star to the vesicle pair after UV illumination. B) Confocal fluorescence microscopy images of two vesicles containing azo-PC before (red) and during (green) UV exposure. The increase in membrane area during UV exposure drives a transition from shallow to deep wrapped (left column) and from deep to fully wrapped state (right column). Scale bars are 10 and 5 μm for the left and right column, respectively.

Equation 7 and Figure 4A also predict that decreasing ν_large_ promotes full wrapping. To test this hypothesis, we incorporated photoresponsive azo-PC lipids into GUV membranes^41^ (Supporting Information Section 1). Upon UV illumination, azo-PC isomerizes, increasing membrane area and lowering ν_large_.^42^ This light-triggered modulation enabled active control of engulfment, driving transitions from shallow to deep, and from deep to full wrapping (Figure 4, Movies S4 and S5), consistent with recent reports on photoswitchable endocytosis of biomolecular condensates.^43^

Repeated UV illumination of the vesicles shown in Figure 4B induced reversible transitions between shallow and deep wrapping, whereas transitions to the fully wrapped state were not reversible (Figure S6). We attribute this asymmetry to the high energetic cost of reopening the membrane neck in combination with a relatively small area change (Supporting Information Section 1). However, further investigation of this asymmetry is warranted to fully understand the underlying mechanisms, as repeated illumination often led to the formation of narrow internal membrane tubes that consumed the membrane area and drove the vesicles toward more spherical shapes.

While the majority of the experimental data conform to the predicted transition, one data point near ϕ ≈ 0.035 remained partially wrapped despite satisfying ν_γ_ < 1. Comparison with a neighboring fully wrapped vesicle reveals the difference lies in their *R*_small_/*L* value: *R*_small_/*L* = 7.3 for the fully wrapped vesicle versus 3.0 for the partially wrapped one. This suggests that the outlier lies near the edge of the geometry-dominated regime, where additional factors such as bending energy may inhibit full engulfment.

## Adhesion Constrained Engulfment

Having established that vesicle geometry primarily dictates the engulfment transition in the *R*_small_/*L* ≫ 1 regime, we now consider the regime where *R*_small_/*L* ≈ 1. Here, the vesicle geometry is insufficient to predict the wrapping outcome. Although full engulfment can still occur when ν_γ_ < 1, the interplay between bending rigidity and adhesion introduces additional constraints. As a result, full wrapping may be suppressed by the deformability of the small vesicle (ν_small_).

Figure 5 shows the full engulfment transition as a function of *R*_small_/*L* (or equivalently $\mathrm{w̃}=wR_{small}^{2}/\kappa$ ) and ν_γ_. We fix ν_large_ = 0.75 and examine three values of ν_small_: 0.8, 0.95, and 0.99, while varying ν_γ_ by changing ϕ (see eq 7). The partial to full wrapping transition is calculated from the total energy curves as a function of *f* (Figure S7). The results show that full engulfment requires an increasing adhesion strength as ν_small_ decreases, illustrating that increased deformability inhibits complete wrapping. This behavior is consistent with prior findings for planar membranes and volume-unconstrained vesicles.^19,22^ Notably, the two experimental points discussed previously, both at ν_γ_ = 0.93 but with *R*_small_/*L* values of 7.3 and 3.0, fall into the fully and partially wrapped regimes, respectively, consistent with their observed wrapping states.

**Figure 5:**
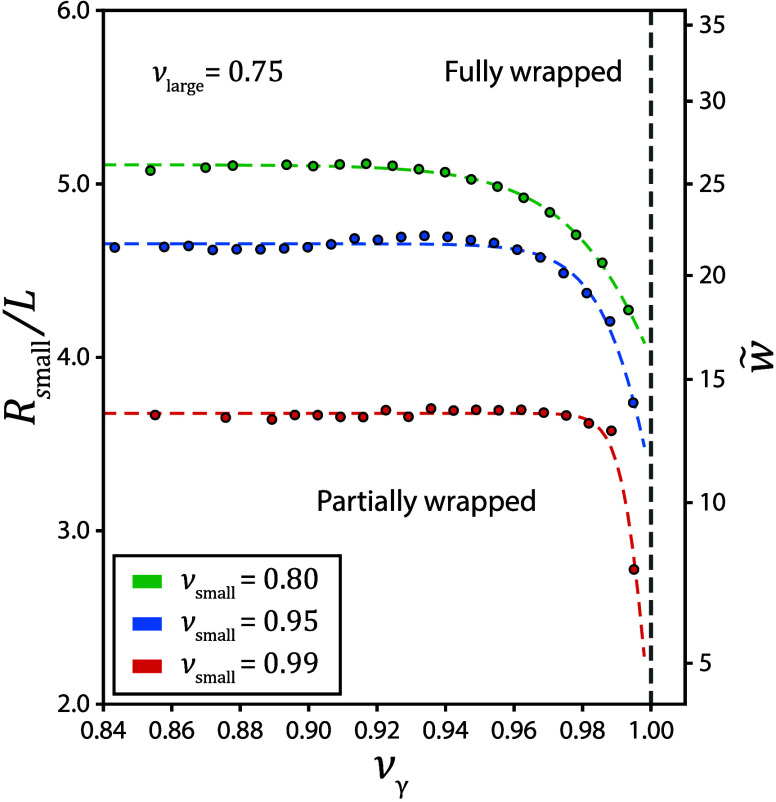
Partial to full wrapping transition as a function of *R*_small_/*L* (left axis), or equivalently $\mathrm{w̃}=wR_{small}^{2}/\kappa$ (right axis), and ν_γ_. The transition is shown for constant ν_large_ = 0.75, while ν_small_ is varied: 0.8 (green), 0.95 (blue), and 0.99 (red). The vertical gray dashed line indicates the geometric cutoff at ν_γ_ = 1, beyond which full engulfment cannot occur.

For all ν_small_, the transition curves show a plateau at low ν_γ_, followed by a steep decline as ν_γ_ → 1. In the absence of spontaneous curvature, the bending energy is scale-independent. Consequently, the bending energy preengulfment depends only on the vesicle shape and is independent of ϕ and ν_γ_. In contrast, as ν_γ_ increases, the large vesicle becomes more spherical after engulfing the small vesicle, reducing the bending energy cost and lowering the adhesion required for full wrapping. This can be observed in the results shown in Figure S7. The effect is strongest near ν_γ_ = 1, where the bending energy gain is largest, and is consistent with previous theoretical studies.^17^ Figure S7 further compares the influence of ν_small_ on this effect. For ν_small_ = 0.8, the wrapping energy curves change little with ν_γ_, giving a gradual decrease in the adhesion threshold as ν_γ_ → 1. For ν_small_ = 0.99, the curves vary strongly with ν_γ_, producing a sharper decrease in the adhesion threshold.

## Conclusions

In this study, we introduced a biomimetic system of large GUVs engulfing smaller ones to investigate how vesicle deformability affects vesicle–vesicle uptake. Adhesion was mediated by depletion interactions, providing a simplified yet biologically relevant analog of cellular internalization. By selectively placing the depletant inside or outside the GUVs, we could control the directionality of engulfment, observing both endocytic and exocytic pathways in the same system.

Our experimental and theoretical analyses show that engulfment is governed by the interplay of adhesion and bending energies, characterized by the bendocapillary length. When *R*_small_/*L* > 1, wrapping is geometry-dominated: whether the small vesicle is partially or fully engulfed depends primarily on the large vesicle’s excess membrane area, quantified by ϕ, ν_large_, and, to a lesser extent, ν_small_. In contrast, when *R*_small_/*L* ≈ 1, the transition also depends on the deformability of the small vesicle, quantified by ν_small_. More deformable vesicles (lower ν_small_) tend to resist complete wrapping, requiring stronger adhesion to achieve full engulfment.

While the complexity of cellular uptake cannot be captured by a single characteristic length, the concept of a bendocapillary length provides physical insight into how adhesion and membrane bending govern the engulfment of deformable vesicles. For biological membranes with bending rigidities of few tens of *k*_B_ *T*, and EVs ranging from tens of nanometres to several micrometres in size,^44^ adhesion energies spanning weak nonspecific interactions to strong receptor–ligand binding (10^–8^ to 10^–4^ J m^–2^)^45^ yield bendocapillary lengths of 10–1000 nm, comparable to EV dimensions. This suggests that many biological systems operate in a regime where deformability might influence uptake.

This study investigates ν_small_ values ranging from ∼0.6 to ∼1, which is comparable to those observed in EVs^46^ and pleiomorphic viruses such as influenza.^47^ Within this range, we find that vesicle deformability significantly influences engulfment, emphasizing both its biological relevance and the design principles it offers for soft drug delivery carriers.

## Supplementary Material













## Data Availability

The simulation data supporting this study have been deposited on Zenodo and are publicly available at 10.5281/zenodo.17342993. Experimental data are available from the corresponding author upon reasonable request.

## Notes

Materials, methods, additional experimental details, supporting data, and numerical analysis (PDF)
Movie S1: Confocal fluorescence movie illustrating depletion-driven interactions between two vesicles (MP4)
Movie S2: Confocal fluorescence movie illustrating the engulfment transition from the free to the fully wrapped state with ν_small_ = 0.89 (MP4)
Movie S3: Confocal fluorescence movie illustrating the engulfment transition from the free to the fully wrapped state with ν_small_ = 0.98 (MP4)
Movie S4: Confocal fluorescence movie illustrating the light-induced transition from the shallow to the deep wrapped state (MP4)
Movie S5: Confocal fluorescence movie illustrating the light-induced transition from the deep to the fully wrapped state (MP4)

## References

[1] György B., Szabó T. G., Pásztói M., Pál Z., Misják P., Aradi B., László V., Pállinger E., Pap E., Kittel A. (2011). Membrane
vesicles, current state-of-the-art: emerging role of extracellular
vesicles. Cell. Mol. Life Sci..

[2] Arandjelovic S., Ravichandran K. S. (2015). Phagocytosis
of apoptotic cells in homeostasis. Nature Immunology.

[3] Cohen F. S. (2016). How viruses
invade cells. Biophys. J..

[4] Marsh M., Helenius A. (2006). Virus entry: open sesame. Cell.

[5] Allen T. M., Cullis P. R. (2013). Liposomal drug delivery
systems: from concept to clinical
applications. Adv. Drug Delivery Rev..

[6] Guimarães D., Cavaco-Paulo A., Nogueira E. (2021). Design of liposomes as drug delivery
system for therapeutic applications. Int. J.
Pharm..

[7] Bareford L. M., Swaan P. W. (2007). Endocytic mechanisms
for targeted drug delivery. Adv. Drug Delivery
Rev..

[8] Joshi B. S., de Beer M. A., Giepmans B. N., Zuhorn I. S. (2020). Endocytosis of extracellular
vesicles and release of their cargo from endosomes. ACS Nano.

[9] Conner S. D., Schmid S. L. (2003). Regulated portals
of entry into the cell. Nature.

[10] Lipowsky R., Döbereiner H.-G. (1998). Vesicles
in contact with nanoparticles and colloids. Europhys. Lett..

[11] Dasgupta S., Auth T., Gompper G. (2014). Shape and
orientation matter for
the cellular uptake of nonspherical particles. Nano Lett..

[12] van
der Ham S., Agudo-Canalejo J., Vutukuri H. R. (2024). Role of shape in
particle-lipid membrane interactions: from surfing to full engulfment. ACS Nano.

[13] Bahrami A. H. (2013). Orientational
changes and impaired internalization of ellipsoidal nanoparticles
by vesicle membranes. Soft Matter.

[14] Agudo-Canalejo J., Lipowsky R. (2015). Critical particle sizes
for the engulfment of nanoparticles
by membranes and vesicles with bilayer asymmetry. ACS Nano.

[15] Agudo-Canalejo J. (2021). Particle engulfment
by strongly asymmetric membranes with area reservoirs. Soft Matter.

[16] Spanke H. T., Style R. W., François-Martin C., Feofilova M., Eisentraut M., Kress H., Agudo-Canalejo J., Dufresne E. R. (2020). Wrapping of microparticles by floppy lipid vesicles. Phys. Rev. Lett..

[17] Bahrami A. H., Raatz M., Agudo-Canalejo J., Michel R., Curtis E. M., Hall C. K., Gradzielski M., Lipowsky R., Weikl T. R. (2014). Wrapping
of nanoparticles by membranes. Adv. Colloid
Interface Sci..

[18] Yi X., Shi X., Gao H. (2011). Cellular uptake
of elastic nanoparticles. Phys. Rev. Lett..

[19] Yi X., Gao H. (2016). Incorporation of soft
particles into lipid vesicles: Effects of particle
size and elasticity. Langmuir.

[20] Imoto Y., Raychaudhuri S., Ma Y., Fenske P., Sandoval E., Itoh K., Blumrich E.-M., Matsubayashi H. T., Mamer L., Zarebidaki F. (2022). Dynamin is primed at
endocytic sites for ultrafast endocytosis. Neuron.

[21] Tang H., Zhang H., Ye H., Zheng Y. (2016). Wrapping of a deformable
nanoparticle by the cell membrane: insights into the flexibility-regulated
nanoparticle-membrane interaction. J. Appl.
Phys..

[22] Midya J., Auth T., Gompper G. (2023). Membrane-mediated
interactions between
nonspherical elastic particles. ACS Nano.

[23] Satarifard V., Lipowsky R. (2023). Mutual remodeling of
interacting nanodroplets and vesicles. Communications
Physics.

[24] Kusumaatmaja H., Lipowsky R. (2011). Droplet-induced budding
transitions of membranes. Soft Matter.

[25] Anselmo A. C., Zhang M., Kumar S., Vogus D. R., Menegatti S., Helgeson M. E., Mitragotri S. (2015). Elasticity
of nanoparticles influences
their blood circulation, phagocytosis, endocytosis, and targeting. ACS Nano.

[26] Sun J., Zhang L., Wang J., Feng Q., Liu D., Yin Q., Xu D., Wei Y., Ding B., Shi X. (2015). Tunable rigidity of
(polymeric core)-(lipid shell) nanoparticles
for regulated cellular uptake. Adv. Mater..

[27] Ziherl P., Svetina S. (2007). Flat and sigmoidally
curved contact zones in vesicle–vesicle
adhesion. Proc. Natl. Acad. Sci. U. S. A..

[28] Evans E. (1992). Equilibrium
“wetting” of surfaces by membrane-covered vesicles. Advances in colloid and interface science.

[29] Murakami K., Ebihara R., Kono T., Chiba T., Sakuma Y., Ziherl P., Imai M. (2020). Morphologies of vesicle
doublets:
competition among bending elasticity, surface tension, and adhesion. Biophys. J..

[30] Chiba T., Sakuma Y., Imai M., Ziherl P. (2023). Morphology of vesicle
triplets: shape transformation at weak and strong adhesion limits. Soft Matter.

[31] Ramachandran A., Anderson T. H., Leal L. G., Israelachvili J. N. (2011). Adhesive
interactions between vesicles in the strong adhesion limit. Langmuir.

[32] Dimova, R. ; Marques, C. The Giant Vesicle Book; CRC Press, 2019.

[33] Machado S., Mercier V., Chiaruttini N. (2019). Limeseg: a
coarse-grained lipid membrane
simulation for 3d image segmentation. BMC Bioinformatics.

[34] Schindelin J., Arganda-Carreras I., Frise E., Kaynig V., Longair M., Pietzsch T., Preibisch S., Rueden C., Saalfeld S., Schmid B. (2012). Fiji: an open-source platform for biological-image
analysis. Nat. Methods.

[35] Vutukuri H. R., Hoore M., Abaurrea-Velasco C., van Buren L., Dutto A., Auth T., Fedosov D. A., Gompper G., Vermant J. (2020). Active particles induce large shape deformations in
giant lipid vesicles. Nature.

[36] Dinsmore A., Wong D., Nelson P., Yodh A. (1998). Hard spheres
in vesicles:
curvature-induced forces and particle-induced curvature. Phys. Rev. Lett..

[37] Asakura S., Oosawa F. (1954). On interaction between two bodies immersed in a solution
of macromolecules. J. Chem. Phys..

[38] Seifert U., Berndl K., Lipowsky R. (1991). Shape transformations
of vesicles:
Phase diagram for spontaneous-curvature and bilayer-coupling models. Phys. Rev. A.

[39] Kraus M., Seifert U., Lipowsky R. (1995). Gravity-induced
shape transformations
of vesicles. Europhys. Lett..

[40] Agudo-Canalejo J. (2020). Engulfment
of ellipsoidal nanoparticles by membranes: full description of orientational
changes. J. Phys.: Condens. Matter.

[41] Pernpeintner C., Frank J. A., Urban P., Roeske C. R., Pritzl S. D., Trauner D., Lohmuller T. (2017). Light-controlled
membrane mechanics
and shape transitions of photoswitchable lipid vesicles. Langmuir.

[42] Aleksanyan M., Grafmüller A., Crea F., Georgiev V. N., Yandrapalli N., Block S., Heberle J., Dimova R. (2023). Photomanipulation of
minimal synthetic cells: Area increase, softening, and interleaflet
coupling of membrane models doped with azobenzene-lipid photoswitches. Advanced Science.

[43] Mangiarotti A., Aleksanyan M., Siri M., Sun T.-W., Lipowsky R., Dimova R. (2024). Photoswitchable endocytosis of biomolecular condensates
in giant vesicles. Advanced Science.

[44] El
Andaloussi S., Mäger I., Breakefield X. O., Wood M. J. (2013). Extracellular vesicles: biology and emerging therapeutic
opportunities. Nat. Rev. Drug Discovery.

[45] Leckband D., Israelachvili J. (2001). Intermolecular
forces in biology. Q. Rev. Biophys..

[46] Arraud N., Linares R., Tan S., Gounou C., Pasquet J.-M., Mornet S., Brisson A. R. (2014). Extracellular
vesicles from blood
plasma: determination of their morphology, size, phenotype and concentration. Journal of Thrombosis and Haemostasis.

[47] Harris A., Cardone G., Winkler D. C., Heymann J. B., Brecher M., White J. M., Steven A. C. (2006). Influenza virus pleiomorphy characterized
by cryoelectron tomography. Proc. Natl. Acad.
Sci. U. S. A..

