# Supplementary material for: From Shallow to Full Wrapping: Geometry and Deformability Dictate Lipid Vesicle Internalization
Source: Nano Lett. 2025 Nov 5;25(46):16451–8. doi: 10.1021/acs.nanolett.5c04322 (PMC12636076; doi:10.1021/acs.nanolett.5c04322)
Supplement: Supplementary file 1 [file nl5c04322_si_001.pdf]

Supporting information for:

From shallow to full wrapping: geometry and deformability dictate lipid vesicle internalization

Stijn van der Ham,[1] Alexander Brown,[2] Halim Kusumaatmaja,[3,*] and Hanumantha Rao Vutukuri[1,†]

[1]*Active Soft Matter and Bio-inspired Materials Lab, Faculty of Science and Technology, MESA+ Institute, University of Twente, 7500 AE Enschede, The Netherlands*
[2]*Department of Physics, Durham University, Durham, DH1 3LE, UK*
[3]*Institute for Multiscale Thermofluids, School of Engineering, The University of Edinburgh, Edinburgh, EH9 3FB, UK*

This PDF file includes:

Supporting text
Figs. S1 to S7
Legends for Movies S1 to S5

Other supporting materials for this manuscript include the following:

Movies S1 to S5

* halim.kusumaatmaja@ed.ac.uk
† h.r.vutukuri@utwente.nl

S1. MATERIALS AND METHODS

EXPERIMENTAL

Materials

Unless otherwise specified, all chemicals were used as received. The lipids, 1,2-dioleoyl-sn-glycero-3-phosphocholine (DOPC) and fluorescent 1,2-dioleoyl-sn-glycero-3-phosphoethanolamine-N-(lissamine rhodamine B sulfonyl) (ammonium salt) (Liss Rhod PE) in chloroform, and 1-stearoyl-2-[(E)-4-(4-((4-butylphenyl)diazenyl)phenyl)butanoyl]-sn-glycero-3-phosphocholine (azo-PC) in powder form, were obtained from Avanti Polar Lipids (Alabaster, AL). Chloroform (≥99.5%), heavy mineral oil, glucose, and sucrose were purchased from Sigma Aldrich. A solution of 10 wt.-% polyacrylamide (molecular weight: 700 000 g/mol to 1 000 000 g/mol) in water was obtained from Polysciences Inc. All aqueous solutions were prepared using ultrapure Milli-Q® water. Sugar solutions were filtered through a 0.2 µm cellulose filter (VWR).

Lipid stock solutions were prepared by dissolving or diluting the lipids in chloroform to the following final concentrations: 12 mg/mL and 3 mg/mL for DOPC, 0.2 mg/mL for Liss Rhod PE, and 3 mg/mL for azo-PC. All stock solutions were stored at −20 °C until use.

Microscopy measurements were conducted using custom observation chambers fabricated from 24 mm × 50 mm #1.5 cover glasses, purchased from VWR. The chambers were assembled by cutting off 0.5 cm of the base of a 1 mL pipette tip and gluing it onto a cover glass using UV-curable glue (Norland Optical Adhesive 68). The glue was cured by placing the chamber under a 365 nm UV LED (Nichia NVSU233B SMD LED UV, 365 nm, 1450 mW) for several minutes. During measurements, the chamber was sealed with Parafilm®.

Preparation of GUVs

Giant unilamellar vesicles (GUVs) were prepared using a modified droplet transfer method based on Refs. 1, 2. A lipid-in-oil solution (LOS) was prepared by mixing 100 µL of DOPC (12 mg/mL) and 10 µL of Liss Rhod PE (0.2 mg/mL) stock in a 20 mL glass vial. The chloroform was evaporated under a gentle stream of N_2 while rotating the vial to form a thin film of lipids at the bottom. The film was kept under vacuum in a desiccator for 1-2 h to remove residual chloroform. Next, 3 g of heavy mineral oil was added to the vial, resulting in a lipid concentration of 425 µM (0.1 mol % Liss Rhod PE). The mixture was sonicated for 1 h at 40 °C. Finally, to ensure proper dissolution, the LOS was kept overnight in the dark at room temperature.

For the incorporation of azo-PC into the GUVs, the same protocol was followed with two modifications. The lipid composition was adjusted to 80 µL of DOPC (3 mg/mL), 3 µL of Liss Rhod PE (0.2 mg/mL), and 27 µL of azo-PC (3 mg/mL). Additionally, the quantity of mineral oil was reduced to 1.5 g, resulting in a lipid concentration of 225 µM (0.1 mol % Liss Rhod PE and 24 mol % azo-PC).

To prepare the GUVs, 200 µL of LOS was gently layered on top of 500 µL of the outer solution (500 mM glucose) in a 2 mL Eppendorf® tube. In a second 2 mL Eppendorf® tube, 200 µL of LOS and 15 µL of the inner solution were combined. The inner solution was either 500 mM sucrose or 500 mM sucrose with 0.25 wt.-% polyacrylamide, depending on the desired adhesion properties. The solution without polyacrylamide was used when studying endocytic engulfment, while the solution with polyacrylamide was used when studying exocytic engulfment, as it induced adhesion on the inside of the GUVs. The mixture was mechanically agitated on an Eppendorf® tube rack for 10 s to form an emulsion of water-in-oil droplets [3]. Next, 120 µL of the emulsion was gently layered on top of the water-oil column in the first Eppendorf® tube and immediately centrifuged at 200 g for 2 min. After centrifugation, the top oil layer was carefully removed with a pipette, and the remaining GUV solution was left to sediment for 30 min to 60 min before being used for measurements. The resulting vesicles exhibited pronounced membrane fluctuations and non-spherical shapes, indicative of low membrane tension (∼ nN/m) and excess membrane area.

GUV-polymer suspensions and sample preparation

To study endocytic engulfment, 20 µL of the GUV solution, taken from the bottom of the tube, was gently mixed with 20 µL of 0.5 wt.-% polyacrylamide in 500 mM glucose by pipetting the solution up and down several times, resulting in a final concentration of 0.25 wt.-% polyacrylamide in the outer solution. To investigate exocytic engulfment, where the inner solution of the GUVs contained 0.25 wt.-% polyacrylamide, the GUV solution was instead mixed with 20 µL

of 500 mM glucose without polyacrylamide. Subsequently, 10 µL of either mixture was added to a custom imaging chamber and sealed with Parafilm® to prevent evaporation. A new chamber was used for each experiment.

Since exocytic engulfment involves a GUV wrapping around an internal vesicle, it requires a configuration in which a large GUV encapsulates a smaller one (an example without adhesion is shown in Fig. S1). Although such vesicle-in-vesicle configurations are less common with our droplet transfer method, they can be reliably generated using microfluidic approaches based on double emulsion techniques [4].

Confocal microscopy imaging

Microscopy measurements were conducted using a confocal laser scanning microscope (Nikon Eclipse Ti-U inverted microscope with a VTinfinity3 CLSM module, Visitech), equipped with a Hamamatsu ORCA-Flash4.0 CMOS camera and a 100x, 1.49 NA oil objective lens. Liss Rhod PE labelled GUVs were excited with a 561 nm laser. Time-lapse imaging was performed at a frame rate of 10 fps. Z-scans were conducted with a step size of 0.25 µm and an exposure time of 50 ms, resulting in a scan rate of approximately 8 slices per second.

Area expansion of azo-PC lipids

Membrane area expansion in GUVs containing 25 mol % azo-PC was achieved by illuminating the sample with an external UV LED (Nichia NVSU233B SMD LED UV, 365 nm, 1450 mW) [5–8]. The UV LED was positioned above the condenser lens of the confocal microscope and focused using an additional lens mounted on a custom optical rail. A low pass filter was added to the UV light path to filter out higher wavelengths. Illumination with UV light resulted in increased GUV membrane area, evidenced by significant membrane fluctuations and shape deformations, indicating a trans-to-cis transition of the azo-PC lipids [5]. However, simultaneous imaging with the 561 nm laser suppressed this expansion. Turning off the UV LED while continuing illumination with the 561 nm laser induced the opposite transition (cis-to-trans), as shown by rapid GUV membrane shrinkage. To minimize the influence of the 561 nm laser and maximize area expansion, time-lapse imaging was performed at low laser power with a frame rate of 500 ms per frame.

We estimate the membrane area increase to be approximately 3% based on comparison with other vesicle-vesicle morphologies. This is lower than expected given the molar ratio of azo-PC and area expansions reported in literature [5]. We suspect that the droplet transfer method causes the fraction of azo-PC incorporated in the GUVs to be lower than intended [9]. Nonetheless, the light modulation effectively demonstrates the significance of excess membrane area, and allows for control over the wrapping configuration.

Analysis

The volume and surface area of the GUVs were determined from confocal z-scans using the LimeSeg plugin in Fiji (ImageJ) [10, 11]. LimeSeg, a particle-based active contour method, segments 3D objects by detecting their outlines, such as those of fluorescently labelled GUVs.

The z-spacing of the z-stacks was corrected by a factor of 0.83 to account for spherical aberration caused by imaging in an aqueous medium with an oil objective lens. This correction factor, obtained from Ref. 12, was calculated using the corresponding ImageJ plugin with the following parameters: numerical aperture (NA) = 1.49, imaging medium refractive index = 1.33, and immersion oil refractive index = 1.52. The accuracy of the correction factor was validated using spherical GUVs (see Fig. S2).

Calibration of L_{exp} using curvature matching

The experimental bendocapillary length, L_{exp}, is constant for a given polymer concentration and can, in principle, be estimated from $L = \sqrt{\frac{\kappa}{w}}$. Assuming a bending rigidity of $\kappa \approx 25\,k_{\mathrm{B}}T$, typical for DOPC membranes [13], and estimating the adhesion strength as $w = 2R_{\mathrm{G}}nk_{\mathrm{B}}T \approx 180\,k_{\mathrm{B}}T/\mu\mathrm{m}^2$, we obtain a theoretical value of $L_{\mathrm{exp}} \approx 0.37\,\mu\mathrm{m}$ for 0.25 wt.% polyacrylamide with a radius of gyration $R_{\mathrm{G}} \approx 45\,\mathrm{nm}$ [14]. Here, the adhesion energy is expressed $E_{\mathrm{ad}} = n\Delta V k_{\mathrm{B}}T = wA_{\mathrm{c}}$ [15], where n is the polymer number density, $\Delta V = A_{\mathrm{c}}d$ is the excluded volume reduction with contact area A_{c} and interaction range d, and $w = ndk_{\mathrm{B}}T$ is the effective adhesion strength per unit area. This estimate, however, assumes contact adhesion between membranes and does not account for effects such as polymer flexibility [16], polydispersity, or membrane fluctuations [17, 18], which can all reduce the effective adhesion strength.

Therefore, to obtain a more accurate estimate of L_{exp}, we compared the curvature profiles of five experimentally observed vesicles (see Fig. S3B) with those generated in simulations using varying values of the simulated bendocapillary length L_{sim}. We expect the best agreement when the ratio R_{small}/L matches between experiment and simulation, which yields the condition:

$$L_{\mathrm{exp}} = \frac{R_{\mathrm{small,\,exp}}}{R_{\mathrm{small,\,sim}}} \cdot L_{\mathrm{sim}}. \tag{S1}$$

Confocal fluorescence z-stacks of each vesicle pair were imported into FIJI (ImageJ) and segmented into 3D meshes using the LimeSeg plugin [10, 11]. The resulting meshes were exported from FIJI as point clouds and processed with a custom Python script, which reconstructed the 3D surface mesh using the Open3D library.

Each vesicle pair was then replicated in simulations using the experimentally measured dimensionless parameters: ϕ, ν_{large}, and ν_{small} as input, while L_{sim} was treated as a tuneable parameter. The simulated vesicle shapes were converted into point clouds and processed using the same in-house Python script as for the experimental data. To ensure consistency in mesh resolution and eliminate potential biases, the simulated meshes were downsampled using a voxel-based approach to match the resolution of the experimental meshes.

For each experimental vesicle pair, we measured the peak mean curvature M^* of the large vesicle, which typically occurs near the contact line and exhibits rotational symmetry around the z-axis defined by the line connecting the vesicles' centres of mass (Fig. S3A). The peak mean curvature M^* was extracted from both experimental and simulated meshes using an identical analysis. First, the local mean curvature $M = \frac{1}{2}(c_1 + c_2)$, where $c_1 = r_1^{-1}$ and $c_2 = r_2^{-1}$ are the principal curvatures, was computed at each vertex of the large vesicle mesh via quadric surface fitting over the local vertex neighbourhood using the libigl library [19]. Representative visualizations of the 3D mesh and the corresponding colour-coded curvature map for vesicle *Endo 02* are shown in Fig. S3C,E.

Next, 9 cross-sections were extracted by intersecting the mesh with planes rotated about the z-axis in 20° increments. From each cross-section, the curvature profile along the intersection curve was computed. The two local peaks in each profile were extracted (marked with red stars in Fig. S3D,F), yielding 18 curvature values per vesicle. These values were averaged to obtain a single representative curvature measurement for the experimental vesicle, M^*_{exp}. The same procedure was applied to the simulated meshes to compute a series of M^*_{sim} values corresponding to different L_{sim}.

For each vesicle pair, the value of M^*_{exp} was compared to $M^*_{\mathrm{sim}}(L_{\mathrm{sim}})$. The L_{sim} that best matched the experimental data was identified by linear interpolation (Fig. S3G). Finally, to obtain L_{exp}, L_{sim} was multiplied by $R_{\mathrm{small,\,exp}}/R_{\mathrm{small,\,sim}}$, where $R_{\mathrm{small,\,sim}}$ was typically set to 1 (Fig. S3H). Averaging across five vesicle pairs yielded $L_{\mathrm{exp}} = 0.6 \pm 0.1\,\mu\mathrm{m}$. In Fig. S3I, slice comparisons between the experimentally measured vesicles and those simulated using Surface Evolver are shown. The parameters for these vesicle pairs are shown in Table S1.

**Vesicle**	ν_{large}	ν_{small}	ϕ	$\mathbf{R}_{\mathrm{small,\,exp}}$ (µm)	$\mathbf{L}_{\mathrm{sim}}$
Endo 01	0.91	0.95	0.016	2.44	0.25
Endo 02	0.90	0.94	0.059	3.51	0.17
Endo 03	0.94	0.90	0.255	7.27	0.08
Exo 01	0.92	0.92	0.019	1.60	0.38
Exo 02	0.92	0.97	0.047	3.42	0.18

TABLE S1. Vesicle parameters corresponding to the slice comparisons shown in Fig. 1H and Fig. S3I for $L_{\mathrm{exp}} = 0.6\,\mu\mathrm{m}$. In simulations, the bending rigidity κ_{sim} and the small vesicle radius $R_{\mathrm{small,\,sim}}$ were consistently set to 1. The adhesion strength is then calculated as $w_{\mathrm{sim}} = \kappa_{\mathrm{sim}}/L^2_{\mathrm{sim}}$.

NUMERICAL

Free energy

For the simulations, we use a variation of the Helfrich free energy model for both the large and small vesicles. The total energy of the system is given by,

$$\begin{aligned} E = &\sum_{\substack{i=\mathrm{small,}\\ \mathrm{large}}} \left(\int_{A_i} \mathrm{d}S_i \left[2\kappa M^2\right] + \sigma_i A_i + P_i V_i \right) \\ &- w \int_{A_c} \mathrm{d}S, \end{aligned} \tag{S2}$$

where A_i and V_i (i = small, large) represent the membrane area and the volume of the small and large vesicles. The first term in the summation accounts for the curvature energy, where κ is the bending rigidity and M the mean curvature of the membrane. We assume zero spontaneous curvature. The other two terms ensure a constant membrane area and volume, where σ_i and P_i are the Lagrange multipliers for the membrane area and the volume constraints, respectively. This reflects experimental conditions, where osmotic conditions constrain GUV volume, while the high area compressibility modulus of lipid membranes ($200\,\mathrm{mN\,m^{-1}}$) ensures negligible area changes under the weak adhesion interactions studied here ($O(10^2)\,k_\mathrm{B}T/\mu\mathrm{m}^2$). The final term represents the adhesion energy, with w denoting the adhesion strength due to depletion interaction and A_c the contact area between the vesicles.

In the case of full wrapping, the membrane of the bud that encapsulates the small vesicle remains connected to the large vesicle through a narrow catenoidal membrane neck. Since resolving the neck region is expensive computationally and the catenoidal neck has zero bending energy, we have used an approximation where we model the encapsulated small vesicle and the remainder of the large vesicle as two separate bodies, without explicitly modelling the catenoid-shape neck. We justify this approximation quantitatively in Fig. S4.

Energy minimisation

In order to find the minimum free energy configurations, we employ the software package Surface Evolver (SE) package version 2.72k, which was obtained directly from Ken Brakke [20]. Example Surface Evolver scripts and output files are deposited on Zenodo (https://doi.org/10.5281/zenodo.17342993). The membrane surfaces of both the large and small vesicles are discretised using a triangulated mesh.

We calculate the mean curvature using the Surface Evolver Method ‘star_perp_sq_mean_curvature’. We specify which facets belong to which vesicle and calculate the mean curvature at each vertex as

$$M_v = \frac{3}{2}\frac{\nabla A_v \cdot \nabla V_v}{\nabla A_v \cdot \nabla A_v}, \tag{S3}$$

where ∇A_v is the area gradient at a vertex v and ∇V_v is the volume gradient at a vertex v. Then, the total bending energy of the system is given by

$$E_b = 2 \sum_{\substack{i=\text{small,}\\ \text{large}}} \sum_{v} \frac{A_{iv}\kappa_i}{3} M_{iv}^2, \tag{S4}$$

where we have summed over each vertex on each vesicle. We assume both vesicles have equal membrane bending constants, $\kappa_\mathrm{small} = \kappa_\mathrm{large} = \kappa$. The areas of the vesicle are held constant by using the facet area method in Surface Evolver, which employs the tension as a Lagrange multiplier. We also use a level constraint of $z = 0$ on the three-phase contact line to restrict the rotational and translational degrees of freedom.

S2. GRAVITY INDUCED VESICLE DEFORMATION

We note that minor discrepancies between experiments and simulations can be seen for some of the large vesicles, for example the smallest volume ratio in Fig. 2A,B (left-most panels) and the fully wrapped regime of Fig. 3A of the main manuscript. In experiments, the large vesicle adopts a flattened, pancake-like shape with a circular cross-section, whereas simulations predict a more elongated, prolate form. This discrepancy can be attributed to gravitational effects that are present in experiments, caused by a density mismatch between the inner and outer sugar solutions (see Supporting Text Section 1). To quantify these gravitational effects, we estimate the dimensionless gravity parameter $g = g_0 \Delta\rho R_{\mathrm{large}}^4/\kappa$ [21], where $g_0 = 9.81\,\mathrm{m\,s^{-2}}$ is the gravitational acceleration, $\Delta\rho \approx 30\,\mathrm{kg\,m^{-3}}$ denotes the density difference between the inner (500 mM sucrose) and outer solution (500 mM glucose), and $\kappa \approx 25\,k_{\mathrm{B}}T$. The large vesicles of the fully wrapped endocytic and exocytic state have radii R_{large} that are larger than 8.6 µm, such that we find $g \geq 16$ for $R_{\mathrm{large}} \geq 8.6\,\mu\mathrm{m}$. This value is in line with the gravity induced flattening that is predicted in Ref. 21, thus confirming that the shape discrepancy is a size-dependent gravitational effect.

S3. DETERMINATION OF WRAPPING FRACTION, CONTACT LINE RADIUS, AND ASPECT RATIO

The wrapping fraction ($f = A_c/A_{small}$), contact line radius (R_c), and small-vesicle aspect ratio (AR) were determined from the 3D point clouds of both experimental and simulated vesicles. Confocal fluorescence z-stacks of each vesicle pair were segmented into 3D meshes using the LimeSeg plugin in FIJI (ImageJ) [10, 11]. The resulting meshes were exported as point clouds and processed with a custom Python script. Each vesicle pair was replicated in simulations using the experimentally measured dimensionless parameters (ϕ, ν_{large}, ν_{small}) and the experimentally determined bendocapillary length ($L_{exp} = 0.6\,\mu m$), as given in Table S2. The simulated vesicle shapes were converted into point clouds and analyzed using the same Python script as for the experimental data. The simulated wrapping fraction was obtained directly from the simulated mesh without further processing.

A. Wrapping fraction

The wrapped region of the experimental small vesicle was identified from point-wise distances to the large vesicle point cloud. Points in the small vesicle point cloud that were within a cut-off distance of at least one point on the large vesicle were classified as wrapped. The wrapping fraction was then computed as the ratio of wrapped surface area (green) to total vesicle surface area (red + green) (Fig. S5A,B). To determine the appropriate cut-off distance, the wrapping fraction was plotted as a function of the distance threshold (Fig. S5C). This revealed a sharp rise at small distances, followed by a gradual linear increase due to inclusion of unbound regions. The cut-off distance was therefore set to the upper limit of the sharp rise, corresponding to 6.5 pixels (0.42 µm).

B. Contact line radius and small vesicle aspect ratio

For both experimental and simulated vesicles, 2D cross-sections were obtained by intersecting the point clouds with planes rotated about the z-axis in 10° increments. Within each slice, the contact point was defined as the midpoint between the nearest points on each vesicle, excluding regions within 10 pixels of the opposite vesicle to avoid overlap artifacts. The contact line radius R_c was calculated as the mean radial distance from the z-axis to the contact point across all slices. The small-vesicle aspect ratio (AR) was defined as $\mathrm{AR} = H/2W$, where H is the vesicle height along the z-axis, and W is the maximum radial distance from the z-axis, which was averaged over all cross-sections. See Fig. S5D for a schematic depiction of the contact point, R_c, H, and W for a single cross-section.

C. Vesicle parameters

**Vesicle**	ν_{large}	ν_{small}	ϕ	$\mathbf{R}_{small,\,exp}$ (µm)	$\mathbf{L}_{sim}$
A1	0.94	0.87	0.001	1.06	0.57
A2	0.91	0.95	0.016	2.44	0.25
A3	0.90	0.94	0.059	3.51	0.17
A4	0.94	0.90	0.255	7.27	0.08
A5	0.92	0.92	0.799	11.73	0.05
B1	0.96	0.95	0.002	0.99	0.61
B2	0.92	0.92	0.019	1.60	0.38
B3	0.92	0.97	0.047	3.42	0.18
B4	0.97	0.92	0.104	2.33	0.26
B5	0.96	0.87	0.615	9.66	0.06

TABLE S2. Vesicle parameters corresponding to Fig. 2 in the main manuscript for $L_{exp} = 0.6\,\mu m$. In simulations, the bending rigidity κ_{sim} and the small vesicle radius $R_{small,\,sim}$ were consistently set to 1. The adhesion strength is then calculated as $w_{sim} = \kappa_{sim}/L_{sim}^2$.

ν_{large}	ν_{small}	ϕ	$\mathbf{R}_{\text{small, exp}}$ (µm)	$\mathbf{L}_{\text{sim}}$
0.91	0.71	0.013	2.24	0.27
0.90	0.87	0.023	2.64	0.23
0.95	0.88	0.037	3.16	0.19
0.91	0.95	0.016	2.44	0.25
0.97	0.98	0.017	3.03	0.20
0.87	0.65	0.014	3.41	0.18
0.88	0.80	0.013	2.89	0.21
0.86	0.89	0.018	2.74	0.22
0.77	0.98	0.035	4.35	0.14

TABLE S3. Vesicle parameters corresponding to Fig. 3 for $L_{\text{exp}} = 0.6\,\mu\text{m}$. In simulations, the bending rigidity κ_{sim} and the small vesicle radius $R_{\text{small, sim}}$ were consistently set to 1. The adhesion strength is then calculated as $w_{\text{sim}} = \kappa_{\text{sim}}/L_{\text{sim}}^2$.

S4. EFFECTIVE REDUCED VOLUME FOR GEOMETRICALLY CONSTRAINED ENGULFMENT

The effective reduced volume of a large vesicle after fully engulfing a small vesicle can be expressed as:

$$\nu_\gamma = 3\sqrt{4\pi}\frac{V_{\mathrm{large}} + V_{\mathrm{small}}}{(A_{\mathrm{large}} - A_{\mathrm{small}})^{3/2}}, \tag{S5}$$

We use the following equations:

$$\nu_{\mathrm{large}} = 3\sqrt{4\pi}\frac{V_{\mathrm{large}}}{(A_{\mathrm{large}})^{3/2}} \tag{S6}$$

$$\nu_{\mathrm{small}} = 3\sqrt{4\pi}\frac{V_{\mathrm{small}}}{(A_{\mathrm{small}})^{3/2}} \tag{S7}$$

$$\phi = \frac{V_{\mathrm{small}}}{V_{\mathrm{large}}} \tag{S8}$$

$$\frac{\nu_{\mathrm{large}}}{\nu_{\mathrm{small}}} = \frac{V_{\mathrm{large}}}{V_{\mathrm{small}}}\left(\frac{A_{\mathrm{small}}}{A_{\mathrm{large}}}\right)^{3/2} = \frac{1}{\phi}\left(\frac{A_{\mathrm{small}}}{A_{\mathrm{large}}}\right)^{3/2} \tag{S9}$$

To rewrite Eq. S5 in terms of the dimensionless variables:

$$\begin{aligned}
\nu_\gamma &= 3\sqrt{4\pi}\frac{V_{\mathrm{large}}(1+\frac{V_{\mathrm{small}}}{V_{\mathrm{large}}})}{\left(A_{\mathrm{large}}(1-\frac{A_{\mathrm{small}}}{A_{\mathrm{large}}})\right)^{3/2}} \\
&= \frac{\nu_{\mathrm{large}}(1+\phi)}{\left(1-\left(\phi\frac{\nu_{\mathrm{large}}}{\nu_{\mathrm{small}}}\right)^{2/3}\right)^{3/2}} \\
&= \frac{\nu_{\mathrm{large}}(1+\phi)}{\left(\frac{\nu_{\mathrm{small}}^{2/3}-\phi^{2/3}\nu_{\mathrm{large}}^{2/3}}{\nu_{\mathrm{small}}^{2/3}}\right)^{3/2}} \\
&= \frac{\nu_{\mathrm{small}}\nu_{\mathrm{large}}(1+\phi)}{\left(\nu_{\mathrm{small}}^{2/3}-\phi^{2/3}\nu_{\mathrm{large}}^{2/3}\right)^{3/2}} \\
&= \frac{(1+\phi)}{\left(\nu_{\mathrm{large}}^{-2/3}-\phi^{2/3}\nu_{\mathrm{small}}^{-2/3}\right)^{3/2}}
\end{aligned} \tag{S10}$$

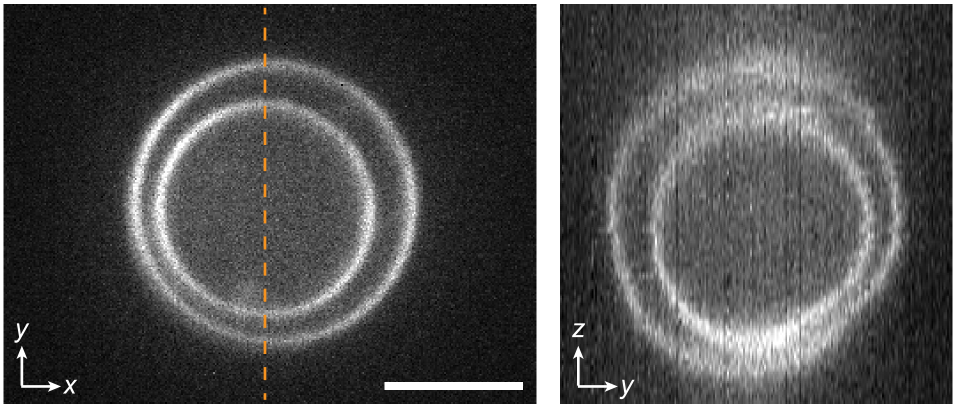

FIG. S1. Confocal microscopy images of a large vesicle with a smaller vesicle in its interior. The measurement was performed in the absence of polyacrylamide. In the left panel the midsection of the vesicle pair in the xy-plane is shown. In the right panel the cross-section in the yz-plane is shown, corresponding to the dotted orange line in the left panel. The scale bar is 5 µm.

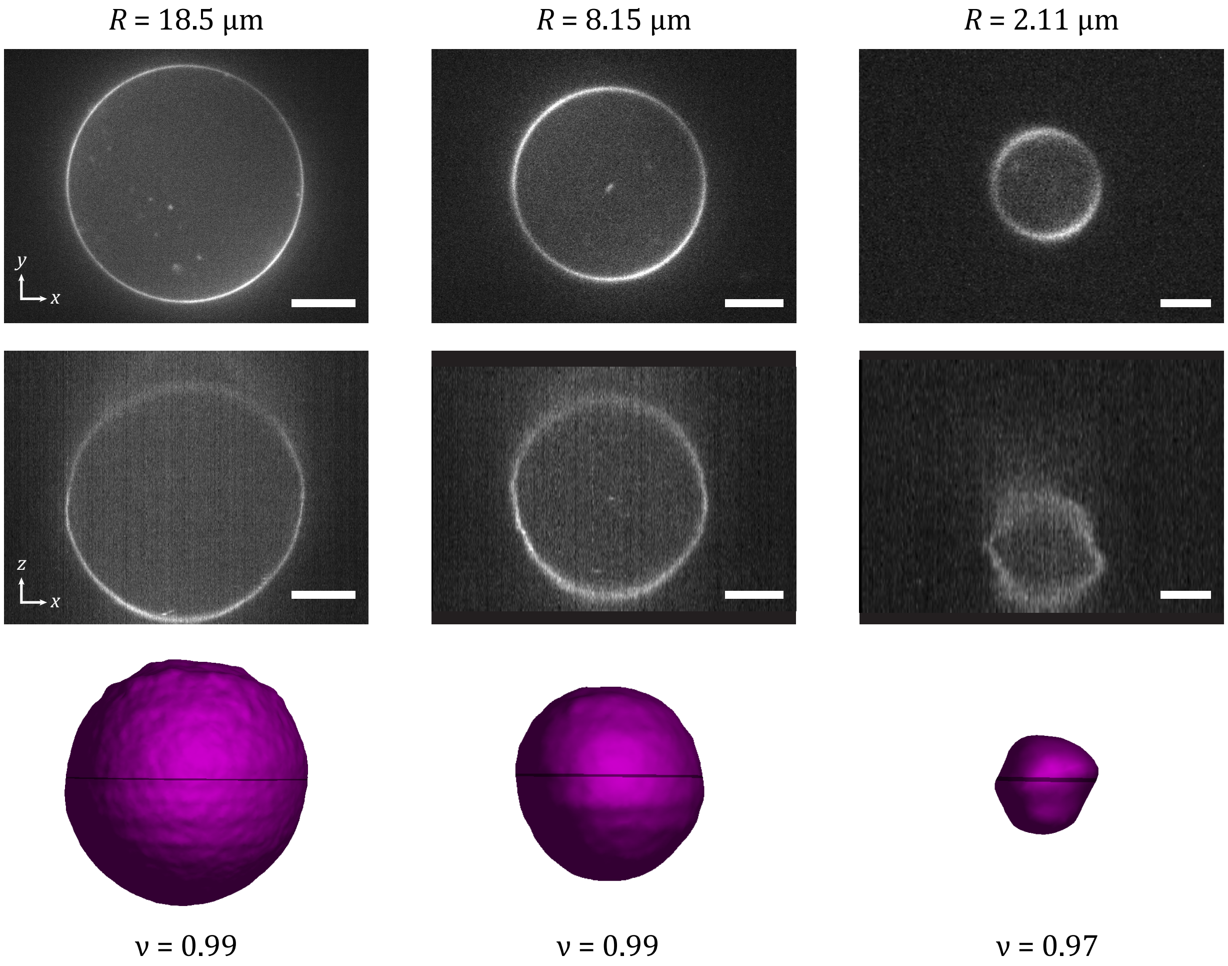

FIG. S2. Accuracy of reduced volume measurements with the LimeSeg plugin in FIJI ImageJ [10, 11] for three different vesicle sizes, given above each column. Vesicles were imaged using fluorescence confocal microscopy. The top row shows the midplane of the vesicle. The middle row shows the vertical cross-section, taken through the middle of the vesicle. The bottom row shows the 3D membrane segmentation from the LimeSeg plugin [11]. Vesicles were prepared in a 100 mM glucose solution and transferred to 70 mM glucose solution to inflate them, which should result in a near perfect spherical shape ($\nu = 1$). Below each column is indicated the reduced volume as determined from the volume and surface area measured with the LimeSeg plugin [11]. Scale bars are 10 μm, 5 μm, and 2.5 μm, for the left, middle, and right column, respectively.

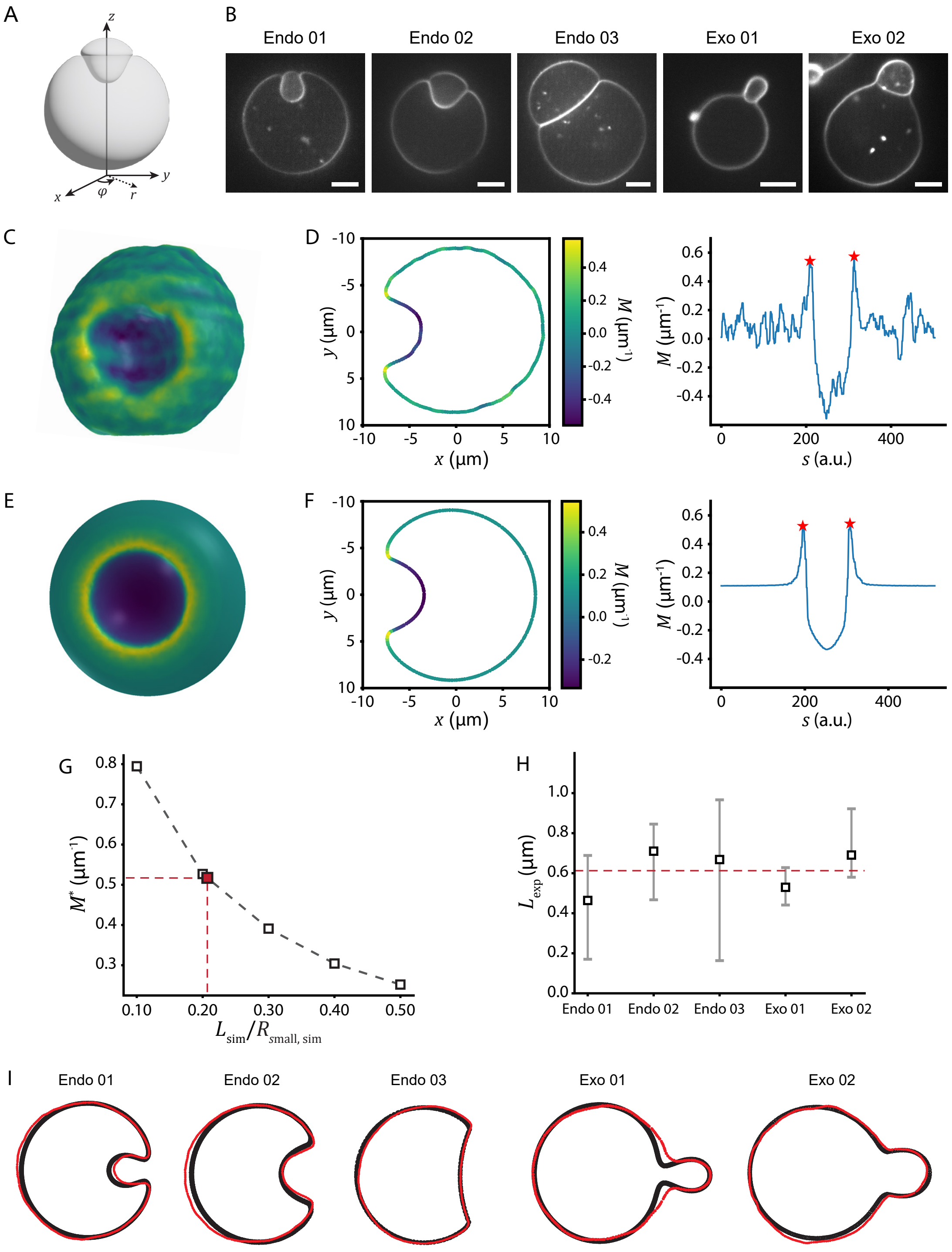

FIG. S3. Calibration of L_{exp} through curvature matching. A) The coordinate system that was used for the curvature analysis. The z-axis is defined as the axis going through the centre of mass of both the small and large vesicle. Intersecting planes had a normal vector perpendicular to the z-axis and were rotated in steps of $\Delta\varphi = 20°$. B) Vesicle pairs that were used for the calibration of L_{exp}. The scale bars are 5 µm. C-F) Peak mean curvature extraction for the experimentally measured (*Endo 02*) (C,D) and simulated (E,F) vesicle at $L_{\mathrm{sim}} = 0.2$. C) and E) show the 3D mesh with the colour coded mean curvature M. D) and F) show a single cross section as a contour plot (left panel) and line plot (right panel). The peak values of M are marked with a red star. G) Linear interpolation of M^*_{exp} (red square) to M^*_{sim} (open squares) as a function of $L_{\mathrm{sim}}/R_{\mathrm{small,\,sim}}$ for *Endo 02*. H) L_{exp} for the 5 analysed vesicles (open squares) and their corresponding error bar, representing the standard deviation, which was determined from the standard deviation in M^*_{exp}. The horizontal red dashed line represents the average L_{exp} value of the 5 measurements points. I) Cross-sectional shape comparison between the experimental (red) and simulated (black) large vesicle morphology for $L_{\mathrm{exp}} = 0.6$ µm.

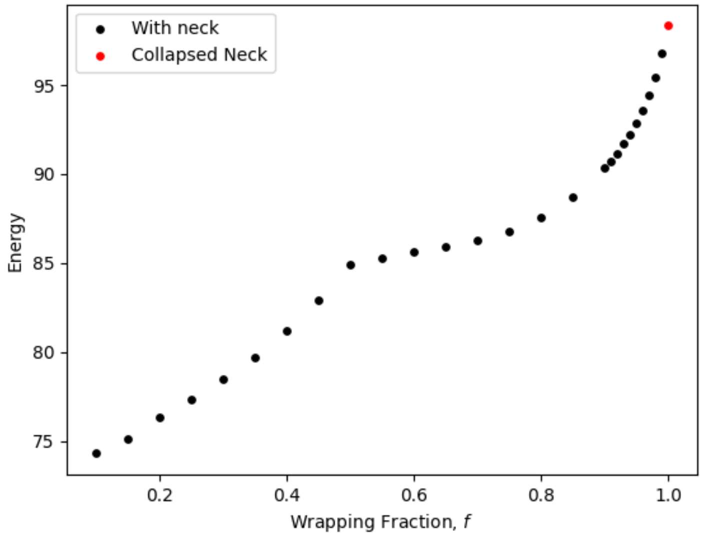

FIG. S4. The total energy as a function of the wrapping fraction for an endocytic engulfment of a small vesicle ($\nu_{\mathrm{small}} = 0.8$) by a large vesicle ($\nu_{\mathrm{large}} = 0.75$). We have set $R_{\mathrm{small}}/L = 0$ and $\phi = 0.04$. For the black data points, the neck is modelled explicitly as the small vesicle is wrapped by the large vesicle. For the red data point, we model the encapsulated small vesicle and the remainder of the large vesicle as two separate bodies, without explicitly modelling the catenoid shape neck. Our results show that the second model (red data point) is the limiting case of the first model (black data points) as the neck shrinks and full wrapping occurs.

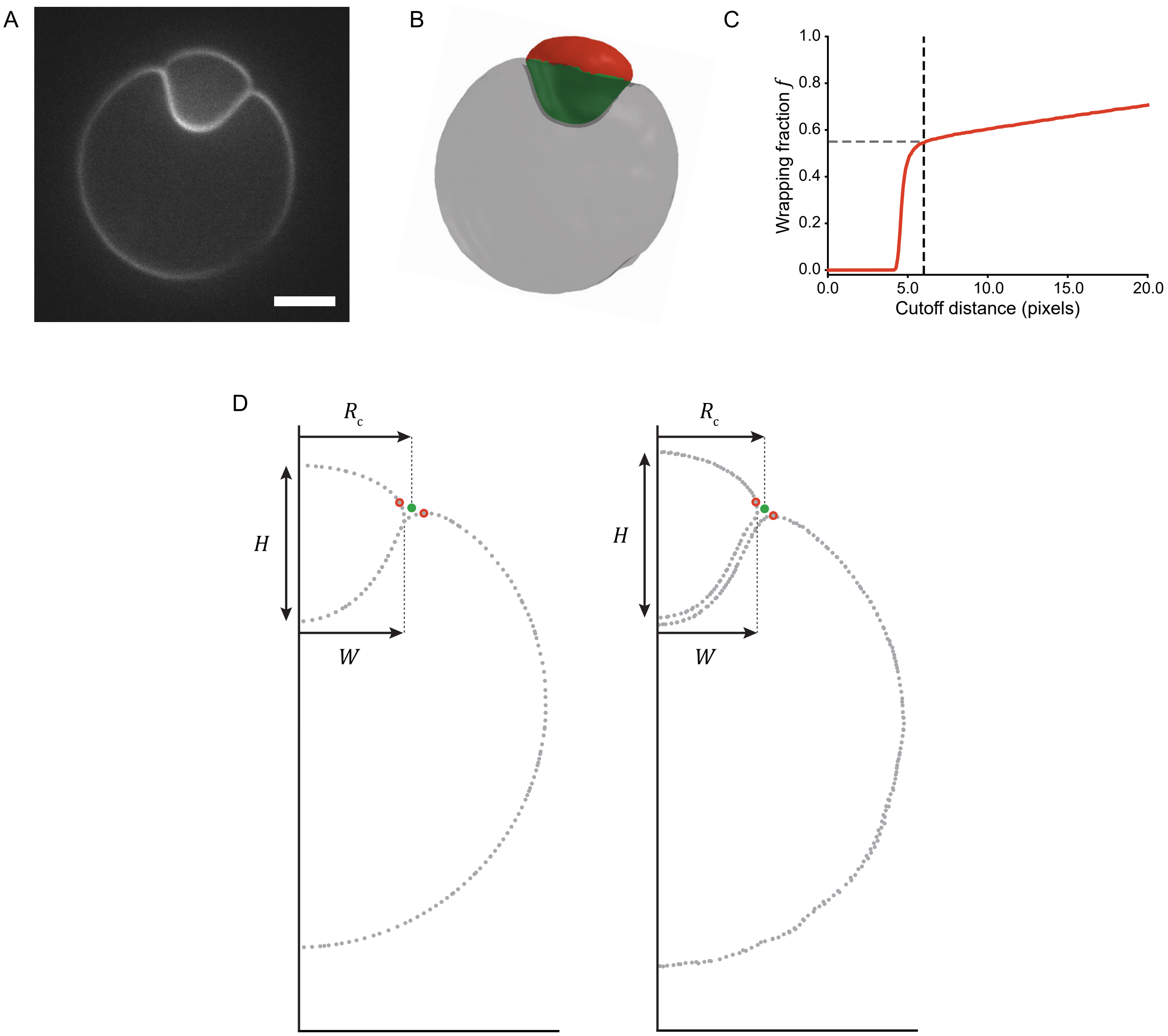

FIG. S5. Example of an experimental measurement of the wrapping fraction. A) Confocal microscopy image of an endocytic vesicle pair. The scale bar is 5 µm B) 3D mesh of the large (grey) and small (green and red) vesicle, where green denotes the wrapped surface and red denotes the unwrapped surface. C) The wrapping fraction as a function of the cut-off distance. The red and green area of the small vesicle shown in panel B were obtained using a cut-off distance of 6.5 pixels (dashed black line). D) Example cross-section illustrating the contact point (green), contact line radius R_c, the vesicle height H, and the maximum radial width W for a simulated (left panel) and experimental (right panel) vesicle pair.

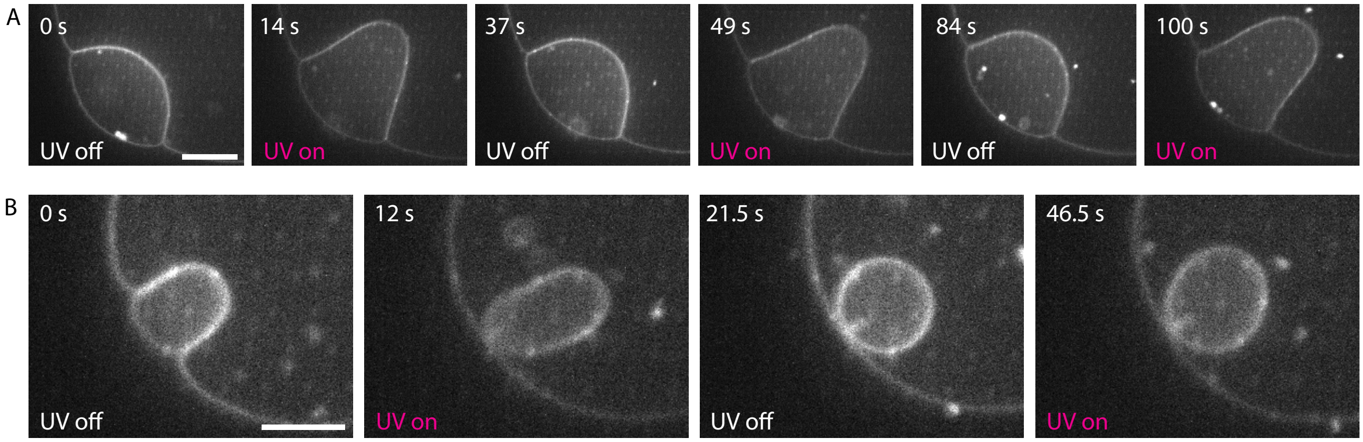

FIG. S6. Confocal fluorescence microscopy time series of two vesicles containing azo-PC during multiple cycles of UV exposure. The increase in membrane area during UV exposure drives transitions between shallow, deep and fully wrapped states. A) The transitions between the shallow and deep wrapped state are reversible over multiple cycles of UV exposure. B) After the initial transition from deep to fully wrapped, the vesicle does not revert back to the deep wrapped state. We believe this asymmetry might originate a combination of the high energy cost associated with reopening the membrane neck and the relatively low area change. Instead, the vesicle remains fully wrapped, assuming a more spherical shape that it had before UV exposure. The latter suggests membrane area is lost to other structures, such as narrow membrane tubes. Scale bars are 10 µm and 5 µm for the top and bottom row, respectively.

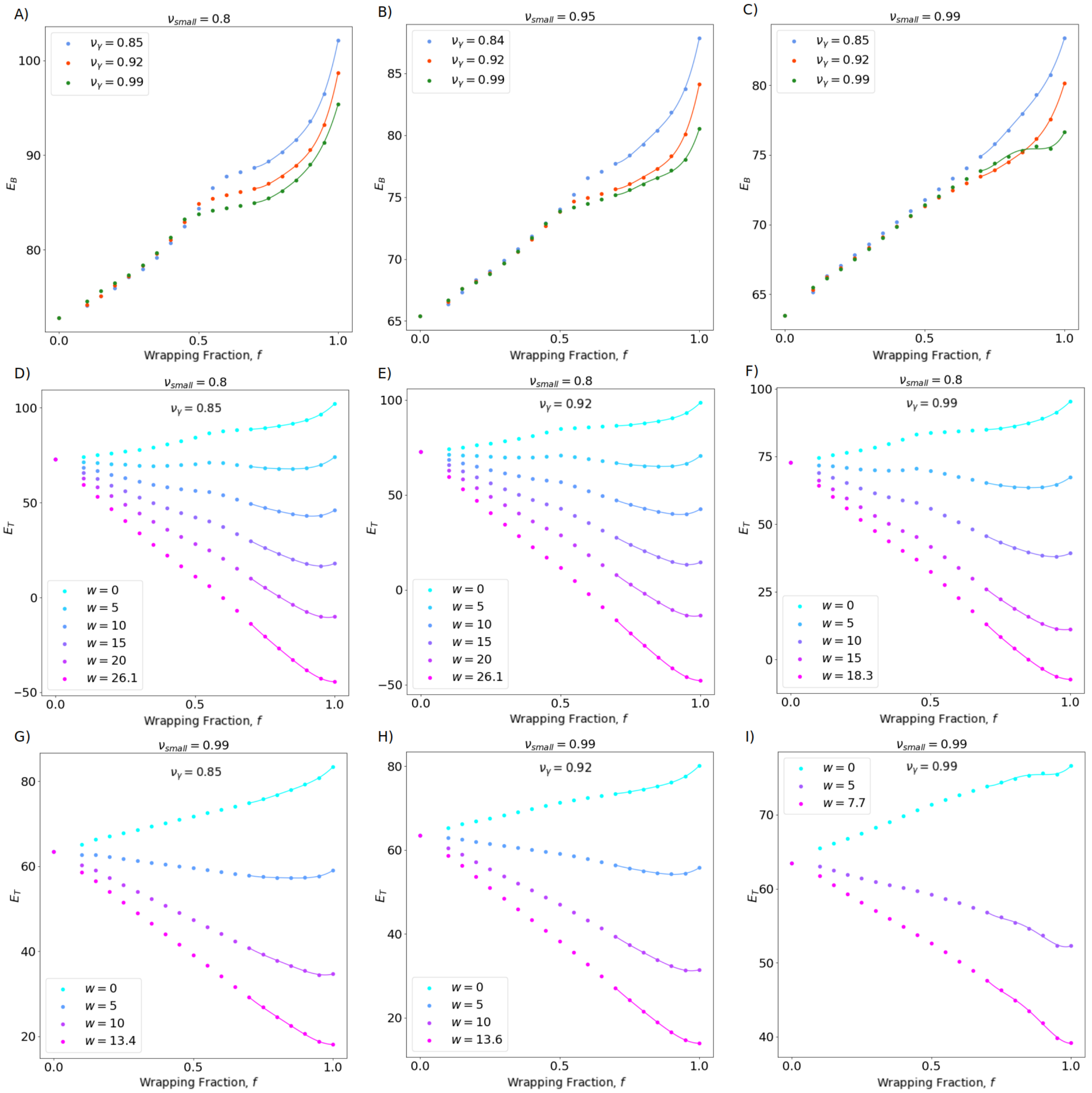

FIG. S7. Energy of the combined large and small vesicle versus the wrapping fraction. The three panels on the first row show the total bending energy E_B for three values of $\nu_{\rm small}$: A) 0.8, B) 0.95, and C) 0.99. In each panel, three ν_γ cases are shown. The two rows below illustrate the calculation of the full wrapping transition. To do this, we plot the total energy by including the contribution from vesicle-vesicle adhesion, given by $E_T = E_B - wA_c$, where w is the adhesion energy per unit area, and A_c is the contact area. Fitting a fourth-order polynomial to the last seven points, we can find the value of w for which the gradient at full wrapping is equal to zero, implying spontaneous full wrapping. In each graph, the largest value of w shown corresponds to the point at which the full wrapping transition occurs. In D)-F), we illustrate this for $\nu_{\rm small} = 0.8$ and varying ν_γ, while in G)-I), the panels are for $\nu_{\rm small} = 0.99$ and varying ν_γ The wrapping energy curves for $\nu_{\rm small} = 0.8$ remain relatively similar across varying ν_γ, resulting in a gradual decrease in the required adhesion as ν_γ approaches unity. In contrast, for $\nu_{\rm small} = 0.99$, the energy curves vary significantly with ν_γ, leading to a sharper drop in the adhesion threshold for complete wrapping.

Supporting Movies:

Movie S1: Confocal fluorescence movie illustrating depletion-driven interactions between two vesicles. The movie shows a transition from the free to the partially wrapped state for two vesicle pairs. The movies were recorded at a rate of 10 fps and are played back at 2x speed (20 fps).

Movie S2: Confocal fluorescence movie illustrating the engulfment transition from the free to the fully wrapped state. The engulfed vesicle had a reduced volume of 0.89. The movie was recorded at a rate of 10 fps and is played back at 2x speed (20 fps).

Movie S3: Confocal fluorescence movie illustrating the engulfment transition from the free to the fully wrapped state. The engulfed vesicle had a reduced volume of 0.98. The movie was recorded at a rate of 10 fps and is played back at 2x speed (20 fps).

Movie S4: Confocal fluorescence movie illustrating the response of GUVs containing azo-PC when subjected to UV light (365 nm). Illumination with UV light causes the smaller vesicle to transition from the shallow to the deep wrapped state. The illumination conditions are indicated in the top right. The movie was recorded at a rate of 2 fps and is played back at 5x speed (10 fps).

Movie S5: Confocal fluorescence movie illustrating the response of GUVs containing azo-PC when subjected to UV light (365 nm). Illumination with UV light causes the smaller vesicle to transition from the deep to the fully wrapped state. The illumination conditions are indicated in the top right. The movie was recorded at a rate of 2 fps and is played back at 5x speed (10 fps).

[1] H. R. Vutukuri, M. Hoore, C. Abaurrea-Velasco, L. van Buren, A. Dutto, T. Auth, D. A. Fedosov, G. Gompper, and J. Vermant, Active particles induce large shape deformations in giant lipid vesicles, Nature **586**, 52 (2020).

[2] S. van der Ham, J. Agudo-Canalejo, and H. R. Vutukuri, Role of shape in particle-lipid membrane interactions: from surfing to full engulfment, ACS Nano **18**, 10407 (2024).

[3] A. Moga, N. Yandrapalli, R. Dimova, and T. Robinson, Optimization of the inverted emulsion method for high-yield production of biomimetic giant unilamellar vesicles, ChemBioChem **20**, 2674 (2019).

[4] N.-N. Deng, M. Yelleswarapu, L. Zheng, and W. T. Huck, Microfluidic assembly of monodisperse vesosomes as artificial cell models, Journal of the American Chemical Society **139**, 587 (2017).

[5] M. Aleksanyan, A. Grafmüller, F. Crea, V. N. Georgiev, N. Yandrapalli, S. Block, J. Heberle, and R. Dimova, Photomanipulation of minimal synthetic cells: Area increase, softening, and interleaflet coupling of membrane models doped with azobenzene-lipid photoswitches, Advanced Science **10**, 2304336 (2023).

[6] J. A. Frank, D. A. Yushchenko, D. J. Hodson, N. Lipstein, J. Nagpal, G. A. Rutter, J.-S. Rhee, A. Gottschalk, N. Brose, C. Schultz, *et al.*, Photoswitchable diacylglycerols enable optical control of protein kinase c, Nature Chemical Biology **12**, 755 (2016).

[7] C. Pernpeintner, J. A. Frank, P. Urban, C. R. Roeske, S. D. Pritzl, D. Trauner, and T. Lohmuüller, Light-controlled membrane mechanics and shape transitions of photoswitchable lipid vesicles, Langmuir **33**, 4083 (2017).

[8] A. Mangiarotti, M. Aleksanyan, M. Siri, T.-W. Sun, R. Lipowsky, and R. Dimova, Photoswitchable endocytosis of biomolecular condensates in giant vesicles, Advanced Science **11**, 2309864 (2024).

[9] H. M. Weakly, K. J. Wilson, G. J. Goetz, E. L. Pruitt, A. Li, L. Xu, and S. L. Keller, Several common methods of making vesicles (except an emulsion method) capture intended lipid ratios, Biophysical Journal **123**, 3452 (2024).

[10] J. Schindelin, I. Arganda-Carreras, E. Frise, V. Kaynig, M. Longair, T. Pietzsch, S. Preibisch, C. Rueden, S. Saalfeld, B. Schmid, *et al.*, Fiji: an open-source platform for biological-image analysis, Nature Methods **9**, 676 (2012).

[11] S. Machado, V. Mercier, and N. Chiaruttini, Limeseg: a coarse-grained lipid membrane simulation for 3d image segmentation, BMC Bioinformatics **20**, 1 (2019).

[12] E. E. Diel, J. W. Lichtman, and D. S. Richardson, Tutorial: avoiding and correcting sample-induced spherical aberration artifacts in 3d fluorescence microscopy, Nature Protocols **15**, 2773 (2020).

[13] H. A. Faizi, C. J. Reeves, V. N. Georgiev, P. M. Vlahovska, and R. Dimova, Fluctuation spectroscopy of giant unilamellar vesicles using confocal and phase contrast microscopy, Soft Matter **16**, 8996 (2020).

[14] J. Francois, D. Sarazin, T. Schwartz, and G. Weill, Polyacrylamide in water: molecular weight dependence of $< \mathrm{R2} >$ and $[\eta]$ and the problem of the excluded volume exponent, Polymer **20**, 969 (1979).

[15] S. Asakura and F. Oosawa, On interaction between two bodies immersed in a solution of macromolecules, The Journal of Chemical Physics **22**, 1255 (1954).

[16] R. Tuinier and H. Lekkerkerker, Excluded-volume polymer-induced depletion interaction between parallel flat plates, The European Physical Journal E **6**, 129 (2001).
[17] W. Helfrich and R. M. Servuss, Undulations, steric interaction and cohesion of fluid membranes, Il Nuovo Cimento D **3**, 137 (1984).
[18] R. Lipowsky and E. Sackmann, *Structure and dynamics of membranes: I. from cells to vesicles/II. generic and specific interactions* (Elsevier, 1995).
[19] A. Jacobson, D. Panozzo, *et al.*, libigl: A simple C++ geometry processing library (2018), https://libigl.github.io/.
[20] K. A. Brakke, The surface evolver, Experimental mathematics **1**, 141 (1992).
[21] M. Kraus, U. Seifert, and R. Lipowsky, Gravity-induced shape transformations of vesicles, Europhysics Letters **32**, 431 (1995).
